# Glycolysis-Related LINC02432/Hsa-miR-98–5p/HK2 Axis Inhibits Ferroptosis and Predicts Immune Infiltration, Tumor Mutation Burden, and Drug Sensitivity in Pancreatic Adenocarcinoma

**DOI:** 10.3389/fphar.2022.937413

**Published:** 2022-06-20

**Authors:** Peng Tan, Mo Li, Zhuoran Liu, Tongxi Li, Lingyu Zhao, Wenguang Fu

**Affiliations:** ^1^ Department of Cell Biology and Genetics / Institute of Genetics and Developmental Biology, School of Basic Medical Sciences, Xi’an Jiaotong University Health Science Center, Xi’an, China; ^2^ Academician (Expert) Workstation of Sichuan Province, The Affiliated Hospital of Southwest Medical University, Luzhou, China; ^3^ Department of General Surgery (Hepatopancreatobiliary Surgery), The Affiliated Hospital of Southwest Medical University, Luzhou, China

**Keywords:** PAAD, ceRNA network, glycolysis, ferroptosis, immune infiltration, tumor mutation burden, drug sensitivity

## Abstract

Pancreatic adenocarcinoma (PAAD) is a malignant cancer with high incidence and mortality. Glycometabolic rearrangements (aerobic glycolysis) is a hallmark of PAAD and contributes to tumorigenesis and progression through numerous mechanisms. This study aimed to identify a novel glycolysis-related lncRNA-miRNA-mRNA ceRNA signature in PAAD and explore its potential molecular function. We first calculated the glycolysis score for each PAAD patient by the ssGSEA algorithm and found that patients with higher hallmark glycolysis scores had poorer prognosis. Subsequently, we obtained a novel glycolysis-related LINC02432/hsa-miR-98–5p/HK2 axis from the TCGA and GEO databases using comprehensive bioinformatics analysis and developed a nomogram to predict overall survival. Furthermore, functional characterization analysis revealed that LINC02432/hsa-miR-98–5p/HK2 axis risk score was negatively correlated with ferroptosis. The tumor immune infiltration analysis suggested positive correlations between ceRNA risk score and infiltrated M0 macrophage levels in PAAD. Correlation analysis found that ceRNA risk scores were positively correlated with four chemokines (CXCL3, CXCL5, CXCL8 and CCL20) and one immune checkpoint gene (SIGLEC15). Meanwhile, tumor mutation burden (TMB), an indicator for predicting response to immunotherapy, was positively correlated with ceRNA risk score. Finally, the drug sensitivity analysis showed that the high-risk score patients might be more sensitive to EGFR, MEK and ERK inhibitors than low-risk score patients. In conclusion, our study suggested that LINC02432/hsa-miR-98–5p/HK2 axis may serve as a novel diagnostic, prognostic, and therapeutic target in PAAD treatment.

## 1 Introduction

Pancreatic cancer is a devastating cancer of the digestive system with poor prognosis, short survival rate, and high mortality (Cai et al., 2021). Pancreatic adenocarcinoma (PAAD) accounts for more than 95% of all pancreatic cancers and is the most common type of pancreatic cancer (Luchini et al., 2021). So far, surgical resection is the only viable curative therapy for PAAD (Tummers et al., 2019). Unfortunately, at least 80% of PAAD patients are originally identified as unresectable or transference tumors. As a result, a substantial number of patients develop either local recurrences or distant metastases after surgical resection. Even with resection, PAAD has a poor prognosis with a 5-years overall survival rate of less than 10% (Yang et al., 2020; Carotenuto et al., 2021). In spite of the tremendous developments in diagnostic tools, surgical approaches, chemotherapy, radiotherapy, and targeted therapy, these approaches can only provide very few survival advantages for PAAD patients (Brero et al., 2020). Clearly, a better understanding of the mechanisms behind PAAD development, is urgently needed to identify novel biomarkers for early diagnosis, prognosis, and treatment.

Whole-genome sequencing shows that roughly 93% of the DNA in the human genome is transcribed into RNA. But, only about 2% of the DNA sequence ultimately encodes a protein. The remaining 98% were called non-coding RNAs (ncRNAs) (Pi et al., 2021). According to the length of the sequences, ncRNAs can be divided into long non-coding RNAs (lncRNAs, over 200 nucleotides) and microRNAs (miRNAs, 19–23 nucleotides in length) (Zhang et al., 2021a). With the deeper research into ncRNA function, the complex regulatory network involving lncRNA, miRNA, and mRNA is drawing increasing research attention worldwide. LncRNAs can act as competitive endogenous RNAs (ceRNAs) to sequester miRNAs from their target mRNAs. This effectively reduces the suppressive effects of miRNAs on mRNA which plays a role in many human diseases, particularly various cancers (Anastasiadou et al., 2018). Abnormal tumor metabolism is increasingly acknowledged as an important hallmark of cancer, leading to renewed interest in therapeutic strategies targeting glycolysis. Studies have identified that the normal pancreas metabolizes glucose through oxidative phosphorylation, whereas PAAD cells prefer aerobic glycolysis for glucose metabolism, known as the Warburg effect. Therefore, exploring the glycolysis-related ceRNA network may offer an attractive new target for prognostic and therapeutic interventions for PAAD.

Ferroptosis, a novel non-apoptotic cell death mode, is closely related to the disturbance of iron-dependent lipid peroxides (Wu et al., 2022). These accumulated lipid reactive oxygen species could lead to ferroptotic cell death. The SLC7A11-GSH-GPX4 signaling axis constitutes the major surveillance system to defend against ferroptosis in cancer cells (Koppula et al., 2022). SLC7A11 and GPX4 are considered as the central regulators of ferroptosis, and SLC7A11 expression and GPX4 activity are always regarded as markers of ferroptosis (Liu P. et al., 2020; Zhang et al., 2021b; Yuan et al., 2021). GPX4 is on the downstream of SLC7A11, utilizing GSH to detoxify lipid peroxides and prevent ferroptosis. SLC7A11 is overexpressed in many cancers, especially in PAAD (Zhou et al., 2021). Research showed that the deletion of SLC7A11 was sufficient to decrease cystine import, downregulate GSH activity, induce tumor ferroptosis, and inhibited PAAD growth (Ping et al., 2022). Studies have shown that, in addition to lipid, lactic acid is also indispensable in the ferroptosis process (Liao et al., 2021). Lactic acid as a glycolytic metabolite has been shown to be a negative regulator of ferroptosis (Zhao et al., 2020). Lactic acid can induce the formation of monounsaturated fatty acids through the HCAR1/MCT1-SREBP1-SCD1 pathway and resist oxidative stress-induced ferroptosis in hepatocellular carcinoma (HCC) cells (Zhao et al., 2020; Liu et al., 2021). It has been suggested that the rerouting of tumour cell metabolism from glycolysis to OXPHOS could make cells more vulnerable to GSH depletion and ferroptosis (Ždralević et al., 2018). Recent studies have shown that ferroptosis is associated with PAAD prognosis and chemotherapy, but its relationship to glycolysis in PAAD remains unclear (Tang et al., 2022). Therefore, this study revealed a new molecular regulatory mechanism of PAAD by investigating the relationship between glycolysis-related ceRNAs and ferroptosis.

The tumor microenvironment (TME) is a complex overall system formed by the association of cancer cells with surrounding stromal and immune cells (Melaiu et al., 2020). It participates in the whole process of tumor occurrence, development, and drug reaction (Li and Wang, 2020; Kumari et al., 2021). PAAD patients have a highly immunosuppressive TME, which is a major cause of immunotherapy resistance in PAAD (Qiu et al., 2021). Accumulation of lactate resulting from aerobic glycolysis forms an acidic environment facilitating tumor invasion, which plays a significant role in shaping the immunosuppressive TME (Lecoultre et al., 2020). Tumor-associated macrophages (TAMs), regulatory T cells (Tregs), and Myeloid-derived suppressor cells (MDSCs) are the principal components of this immunosuppressive TME. These cells have been reported to facilitate systemic T cell dysfunction, allowing PAAD to evade immune detection (Liang et al., 2021; Truong and Pauklin, 2021). Studies have shown that knockdown of interferon-inducible protein 16 (IFI16) significantly enhances gemcitabine treatment in PAAD, which may be associated with reduced TAMs infiltration in the tumor microenvironment (Chen et al., 2021). Recent clinical studies have identified tumor mutation burden (TMB) as an indicator for predicting response to immunotherapy (Zhang W. et al., 2021). High TMB have better response to PD-1/PD-L1 therapy across diverse tumor entities (Ak et al., 2021). Therefore, analyzing the relationship between glycolysis-related ceRNA networks, tumor-infiltrating immunity, TMB and drug sensitivity is critical to explain the potential molecular mechanisms implicated in the development of PAAD, and identify promising biomarkers and novel therapeutic drugs.

In this study, we comprehensively analyzed several databases to construct a novel glycolysis-related LINC02432/hsa-miR-98–5p/HK2 ceRNA network and constructed a three-gene signature using Cox regression survival analysis to forecast the prognosis for PAAD patients. Next, we downloaded ferroptosis-related gene sets (driver, suppressor, and marker) from FerrDb and analyzed the correlation between the LINC02432/hsa-miR-98–5p/HK2 axis risk score and ferroptosis. Meanwhile, we probed the relationship among the ceRNA axis and immune cell infiltration in PAAD by ImmuCellAI and the CIBERSORT algorithm. Furthermore, we explored the correlation between LINC02432/hsa-miR-98–5p/HK2 ceRNA network and the expression levels of 40 known chemokines and eight immune checkpoint genes through Pearson correlation analysis in PAAD cancers. We downloaded somatic mutation data from The Cancer Genome Atlas (TCGA) GDC data to assess the relationship between the ceRNA network and TMB. Finally, the R package oncoPredict and the Genomics of Drug Sensitivity in Cancer (GDSC) database were used to predict potential drugs for PAAD patients with high risk score. In conclusion, a novel glycolysis-related LINC02432/hsa-miR-98–5p/HK2 ceRNA network targeting PAAD patients was constructed and its functions were analyzed, which may establish new insights for clinical decision making and precision medicine.

## 2 Materials and Methods

### 2.1 Gene Expression Profile Data Collection

Download two lncRNA expression arrays (GSE57144 and GSE86436) from the Gene Expression Omnibus (GEO) database (http://www.ncbi.nlm.nih.gov/geo/). The GSE57144 dataset contained three pancreatic cancer tissues and three adjacent normal tissues. The GSE86436 dataset contained six pairs of pancreatic tumor tissues with normal adjacent tissues. Meanwhile, seven mRNA microarray datasets (GSE15471, GSE16515, GSE28735, GSE32676, GSE62165, GSE62452, and GSE71729) were obtained from the GEO database. GSE15471 contained 39 PAAD tumor tissues and 39 adjacent normal controls. GSE16515 included 36 PAAD tumors and 16 adjacent non-tumor tissues. GSE28735 contained 45 PAAD tumors and 45 adjacent non-tumor tissues. GSE32676 included 25 PAAD samples and seven adjacent non-tumor samples. GSE62165 contained 118 PAAD tissues and 13 adjacent control tissues. GSE62452 included 69 PAAD tumors with 61 adjacent non-tumor tissues. GSE71729 contained 145 PAAD tumors and 46 adjacent non-tumor samples. In addition, the level 3 RNA sequencing data (lncRNA, miRNA, and mRNA) and corresponding clinical information for patients with PAAD were obtained from TCGA database by the TCGAbiolinks package of R software (version 4.0.4).

### 2.2 Evaluation of Glycolysis Score

The gene sets associated with the glycolysis pathway (reactome glycolysis and hallmark glycolysis) were obtained from the Molecular Signatures Database (MSigDB, https://www.gsea-msigdb.org/gsea/msigdb). Then, single sample Gene Set Enrichment Analysis (ssGSEA) was used to calculate the glycolysis score for each PAAD patient. We divided PAAD patients into high glycolysis score and low glycolysis score groups based on the median glycolysis score. Finally, the association of glycolysis score with prognosis was assessed by Kaplan-Meier survival analysis.

### 2.3 Identification of Glycolysis-Related Genes

The GEO2R web application (http://www.ncbi.nlm.nih.gov/geo/geo2r) was performed to analyze differentially expressed lncRNAs (DELs) and differentially expressed genes (DEGs) among PAAD tumor tissues and adjacent non-tumor tissues. Meanwhile, we used the R package limma to determine DELs and DEGs among the high glycolysis score and low glycolysis score groups. The log2 (fold change) >= 1.0 or <= -1.0 and *p*-value < 0.05 were set as the cut-off criteria. We used Venn diagram analysis to further screen for glycolysis-related lncRNAs and mRNAs in PAAD. Meanwhile, through Kaplan-Meier analysis, Pearson correlation analysis, and univariate Cox regression analysis, the key prognostic genes of glycolysis-related were identified.

### 2.4 Construction of a Glycolysis-Related lncRNA-miRNA-mRNA ceRNA Network

The downstream miRNAs of the target lncRNAs were predicted by miRNet database (https://www.mirnet.ca/) and starBase database (http://starbase.sysu.edu.cn/index.php). The miRTarBase database (http://mirtarbase.cuhk.edu.cn/) was used to forecast upstream miRNAs of key mRNAs. We selected mRNA-miRNA interactions with strong experimental evidence (reporter analysis or western blot) for further study. Based on ceRNA theory and the association between lncRNAs, miRNAs and mRNAs, ceRNA networks were established by Cytoscape software (version 3.8.0). We calculated the expression correlations of lncRNA-mRNA, lncRNA-miRNA, and mRNA-miRNA pairs by Pearson correlation analysis. We chose gene pairs with |r| > 0.1 and *p*-value < 0.05 for further analysis.

### 2.5 Gene Expression and Subcellular Localization Analysis

We used UALCAN to explore the HK2 protein expression in PAAD with data from the Clinical Proteomic Tumor Analysis Consortium (CPTAC). Moreover, the immunohistochemistry (IHC) staining data of protein expression and distribution of HK2 in PAAD tissues and normal tissues were obtained from the Human Protein Atlas (HPA) database (https://www.proteinatlas.org/). lncLocator (http://www.csbio.sjtu.edu.cn/bioinf/lncLocator/) was used to obtain subcellular localization of LINC02432. We also examined the specificity of SIGLEC15 mRNA expression in different pancreatic single cell types using the single-cell RNA-seq (scRNA-seq) data through the HPA dataset.

### 2.6 Construction and Assessment of ceRNA-Related Prognostic Model

We used multiple Cox regression analysis of the LINC02432/hsa-miR-98–5p/HK2 axis levels to calculate the risk scores in PAAD patients. According to the median risk score, we performed Kaplan-Meier analysis in PAAD patients using the survminer R package. At the same time, we used the ROC package in R to draw receiver operating characteristic (ROC) curves for 1-year, 3-years and 5-years survival rates, and calculated the corresponding area under the curve (AUC) to evaluate the predictive power of ceRNA-related features. Based on Cox proportional hazards regression analysis, we developed a 5-years overall survival risk stratified nomogram based on prognostic factors using the rms library in R.

### 2.7 Correlation Analysis Between Ferroptosis and Glycolysis

A total of 259 ferroptosis-related genes were downloaded from the FerrDb website (http://www.zhounan.org/ferrdb/legacy/index.html), including 108 driver genes, 69 suppressor genes, and 111 marker genes. The ssGSEA heatmap was done using the pheatmap R package to show the enrichment of the three ferroptosis-related gene sets across all PAAD samples. The correlation heatmap was generated by R software with the corrplot function package. The GEPIA2 database (http://gepia2.cancer-pku.cn/) was used to explore the differential expression of important ferroptosis suppressor genes between PAAD and normal tissues. Survival analyses of key ferroptosis suppressor genes were conducted with the Kaplan-Meier plotter (http://kmplot.com/analysis/).

### 2.8 Tumor Immune Infiltration Analysis

The ImmuCellAI and the CIBERSORT algorithm were used to evaluate the abundance of tumor-infiltrating immune cells. We used the ImmuCellAI (Immune Cell Abundance Identifier, http://bioinfo.life.hust.edu.cn/web/ImmuCellAI/) algorithm to forecast the abundance of 24 immune cells from transcriptome data using gene set signatures. Meanwhile, we visualized the 24 immune cell infiltration estimation of TCGA samples as a heatmap by the R package ComplexHeatmap. According to the CIBERSORT algorithm, we selected the LM22 gene signature and 1000 permutation parameters in R to analyze the score of 22 immune cells. LM22 is an annotated gene signature matrix consisting of 547 genes that defines 22 immune cell subtypes and can be downloaded from the CIBERSORT portal (http://cibersort.stanford.edu/). Macrophage lineage expression profiles in LM22 signature were derived from freshly isolated monocytes in peripheral blood monocytes. M0 macrophages were generated by monocyte differentiation in human serum for 7 days. M1 macrophages were generated by monocyte differentiation in colony stimulating factor 1 (CSF1) for 7 days and then stimulated with LPS and IFNγ for 18 h. M2 macrophages were generated by monocyte differentiation in CSF1 for 7 days and then stimulated with IL-4 for 18 h (Newman et al., 2015). The proportions of 22 immune cell infiltrations in PAAD patients were visualized by cumulative histograms using the ggplot2 package. We calculated the Pearson correlation coefficients of ceRNA risk scores and tumor-infiltrating immune cells by the R package ggpubr.

### 2.9 Expression Levels Analysis of Chemokines and Immune Checkpoint Genes

The expression of chemokine and immune checkpoint genes was compared between PAAD tumors and adjacent normal samples by the GEPIA2 database website (http://gepia2.cancer-pku.cn/). We summarized the differential expression of chemokine and immune checkpoint genes among high and low ceRNA risk score groups as boxplots by the ggplot2 package in R software. The clustering analysis of differentially expressed chemokines and heatmap visualization of the correlation matrix were performed using the R corrplot package. The Pearson correlation analysis of ceRNA risk score with the key chemokines and immune checkpoint genes was performed and visualized using the R package ggpubr.

### 2.10 Association Among Somatic Mutation and Risk Score

We used the maftools package in R software to organize the single-nucleotide polymorphism (SNV) data downloaded from the TCGA database in multiple alignment (MAF) format. Meanwhile, we plotted horizontal histograms showing the genes with the highest mutation frequencies by the maftools package in R. The Pearson correlation coefficient between the top 10 frequently mutated genes was calculated and plotted using the corrplot package in R. We assessed TMB values for each sample and analyzed the overall survival in the high and low TMB groups using the Kaplan-Meier method. Meanwhile, we compared TMB values among high- and low risk groups, and assessed the association of TMB with risk scores.

### 2.11 Tumor Immune Dysfunction and Exclusion (TIDE) Analysis in PAAD.

The TIDE (http://tide.dfci.harvard.edu/) model was a computational method, which integrated the expression signatures of T cell dysfunction and T cell exclusion to model tumor immune evasion. The clinical response of immune checkpoint blockade (anti-PDCD1 and anti-CTLA4) could be predicted by TIDE algorithm based on pre-treatment tumor profiles. The TIDE score was compared between high risk score and low risk score groups.

### 2.12 Drug Sensitivity Analysis

Genomics of Drug Sensitivity in Cancer (GDSC, https://www.cancerrxgene.org/) is the largest publicly available pharmacogenomics database which can be used to predict response to anti-cancer drugs. In this study, we used data from GDSC2, an updated version of GDSC containing 809 cell lines and 198 compounds. Based on this database, we used the R software package oncoPredict to predict the antineoplastic drug susceptibility for PAAD patients with the high- and low risk groups. The regression analysis was conducted to obtain the half-maximal inhibitory concentration (IC50) estimated value of each specific antineoplastic drug treatment.

### 2.13 Statistical Analysis

Most statistical analysis was done by the aforementioned bioinformatics tools. The R software (version 4.0.4) was used for all the rest of the statistical analyses. Two-tailed Student’s t test was used to estimate the differential expression levels of mRNA, miRNA, and lncRNA. The *p*-value adjustment was performed by the Benjamini–Hochberg FDR method. The correlation was assessed by the Pearson correlation coefficient. A *p*-value < 0.05 was considered as statistically significant.

## 3 Results

### 3.1 Identification of Glycolysis-Related lncRNAs and mRNAs in PAAD.

**FIGURE 1:**
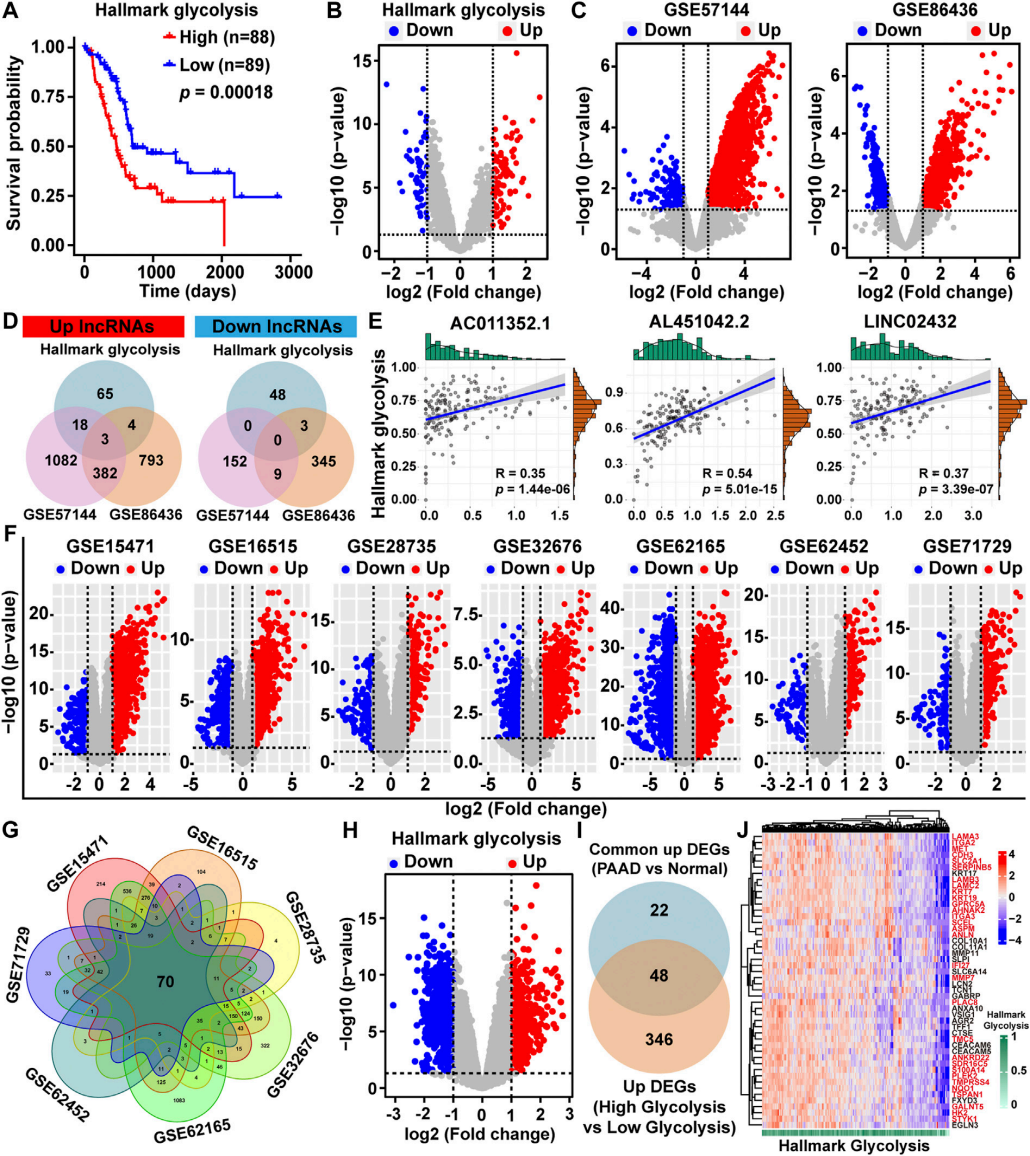
Identification of glycolysis-related differentially expressed lncRNAs and mRNAs. **(A)** Kaplan-Meier survival analysis of PAAD patients based on hallmark glycolysis score. **(B)** Volcano map of the DELs among high-glycolysis and low-glycolysis groups. **(C)** DELs volcano maps between PAAD tissues and normal tissues in the GSE57144 and GSE86436 datasets. Upregulated lncRNAs were indicated by red dots and downregulated lncRNAs were indicated by blue dots. **(D)** Venn diagram depicting the overlap among three sets of DELs. **(E)** The Pearson correlation analysis was performed to evaluate the association between glycolysis ssGSEA score and three lncRNAs. **(F)** Volcano plots of DEGs between PAAD tissues and normal tissues in the GSE15471, GSE16515, GSE28735, GSE32676, GSE62165, GSE62452, and GSE71729 datasets downloaded from the GEO database **(G)** Venn diagram of upregulated DEGs based on the seven GEO datasets. **(H)** DEGs volcano plot among high-glycolysis and low-glycolysis groups. **(I)** The overlapping upregulated DEGs were identified by Venn plot. **(J)** Heat map of 48 upregulated DEGs related to glycolysis.

The flowchart in Supplementary Figure S1 outlines the entire design and process of this research. We first obtained two glycolysis pathway gene sets (hallmark glycolysis and reactome glycolysis) from the MSigDB. Next, a heatmap was drawn to show the glycolytic activity of each pathway in each TCGA-PAAD sample quantified by the ssGSEA algorithm (Supplementary Figure S2A). Kaplan–Meier survival analysis results indicated that the PAAD overall survival was associated with the hallmark glycolysis score (Figure 1A), but was not associated with the reactome glycolysis score (Supplementary Figure S2B). Patients with higher hallmark glycolysis scores had poor prognoses.

To identify glycolysis-related lncRNAs, we first obtained the DELs between high/low hallmark glycolysis score groups (Figure 1B). The volcano maps showed the lncRNA expression patterns between PAAD and normal tissues based on the GEO datasets (GSE57144 and GSE86436) (Figure 1C). To investigate potential overlaps of the three sets of significant DELs, we created a visualization of the overlaps as a Venn diagram. We found three upregulated glycolysis-related lncRNAs (LINC02432, AL451042.2, and AC011352.1), but no downregulated glycolysis-related lncRNAs were found (Figure 1D). Pearson correlation analysis indicated that the expression of LINC02432, AL451042.2, and AC011352.1 were positively associated with glycolytic activity level (Figure 1E).

To further explore DEGs between PAAD and normal samples, we downloaded seven gene microarrays datasets from the GEO database: GSE15471, GSE16515, GSE28735, GSE32676, GSE62165, GSE62452, and GSE71729. The distribution of all DEGs according to the two dimensions of -log10 (*p*-value) and log2 (fold change) were represented by volcano maps in Figure 1F. In the ceRNA network, the correlation of lncRNA and mRNA was positive. Given that the three glycolysis-related lncRNAs (AC011352.1, AL451042.2, and LINC02432) were all found to be upregulated in PAAD, we further identified upregulated DEGs. Among the upregulated DEGs from the seven GEO datasets, we obtained 70 overlapping upregulated DEGs through Venn diagram analysis (Figure 1G). Then, DEGs between high/low hallmark glycolysis score groups were analyzed using the volcano map, of which, 394 were upregulated and 638 were downregulated (Figure 1H). The result of Venn analysis suggested that 48 upregulated DEGs related to glycolysis were identified for further study by overlapping the 70 DEGs and 394 upregulated genes (Figure 1I). In addition, we drew a cluster heat map for 48 upregulated DEGs expressions in Figure 1J. Finally, univariable Cox regression analysis was performed to assess the prognostic impact of 48 upregulated DEGs related to glycolysis (Supplementary Figure S3). The results showed that 30 of the 48 upregulated DEGs (*p*-value < 0.05 and HR > 1.2) were considered key prognostic genes of glycolysis-related. The above 30 genes were marked with red font in Figure 1J. We used the Pearson correlation analysis to determine the associations between three lncRNAs and 30 key mRNAs. We found that the expression of AL451042.2 and LINC02432 was significantly positively correlated with the expression of 30 key mRNAs, while the expression of AC011352.1 was significantly positively correlated with 23 of these genes (Supplementary Figure S4).

### 3.2 Construction and Analysis of Glycolysis-Related ceRNA Network

**FIGURE 2:**
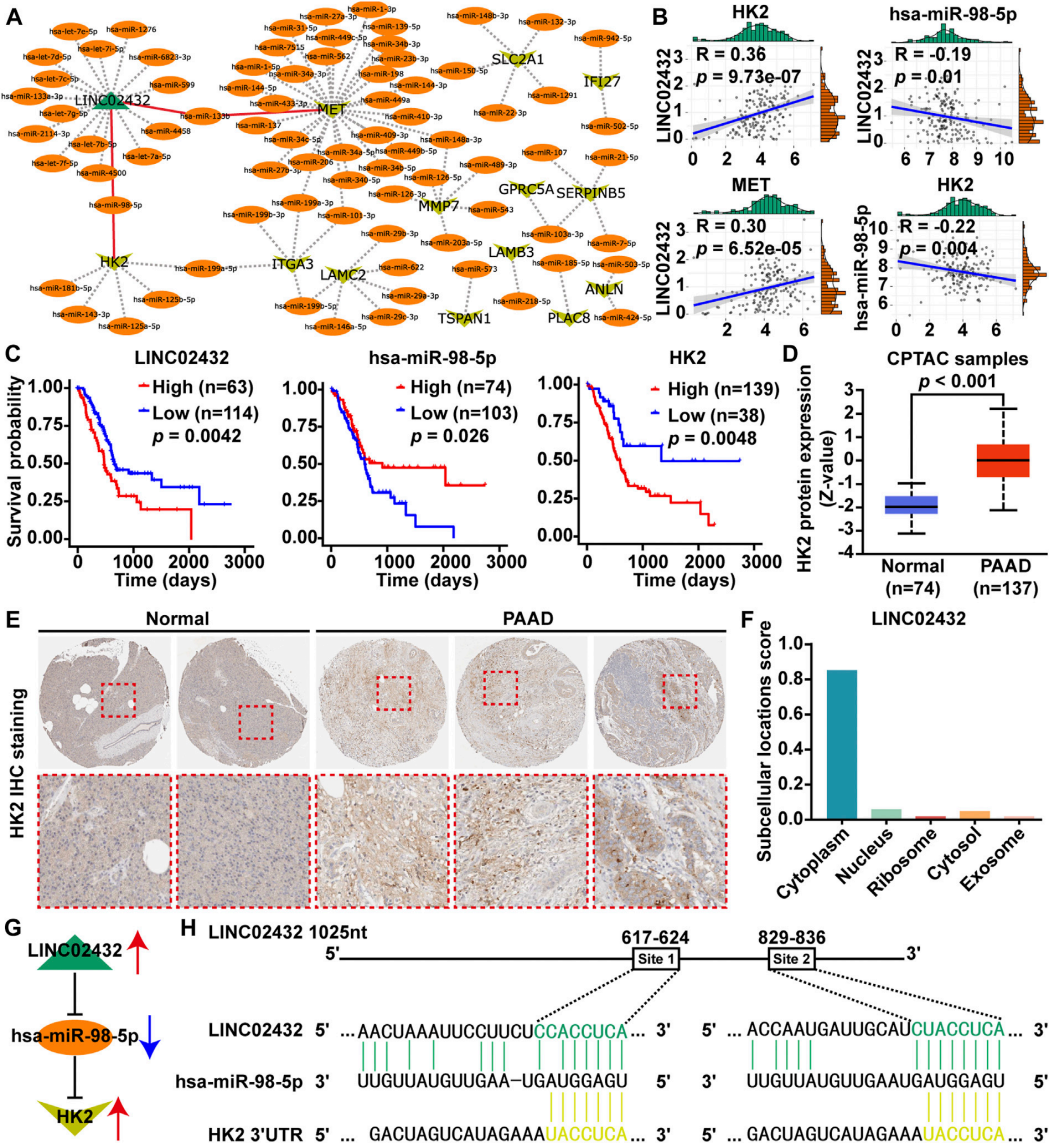
Identification of glycolysis-related ceRNA network modules. **(A)** A diagram of lncRNA-miRNA and mRNA-miRNA interactive networks was constructed by Cytoscape software. **(B)** The Pearson correlation analysis was performed to identify the correlation between genes (LINC02432, HK2, MET, and hsa-miR-98–5p). **(C)** Survival analysis of LINC02432, hsa-miR-98–5p and HK2 was performed using Kaplan-Meier survival curves. **(D)** HK2 protein expression of PAAD patients was evaluated in the CPTAC datasets via the UALCAN database. **(E)** IHC staining of HK2 protein was analyzed in the HPA database. **(F)** Subcellular localization of LINC02432 was predicted using the lncLocator website. **(G)** Schematic illustration of the LINC02432/hsa-miR-98–5p/HK2 ceRNA axis. **(H)** Identification of target sites for hsa-miR-98–5p on the 3′-UTR of LINC02432 and HK2.

Based on the ceRNA network theory, we predicted the downstream potential miRNA that could potentially bind to three upregulated glycolysis-related lncRNAs (AC011352.1, AL451042.2, and LINC02432) using starBase and miRNet. Potential upstream miRNAs targeting 30 key glycolysis-related genes were then analyzed using miRNA-target interactions supported by strong experimental evidence in miRTarBase. Meanwhile, the lncRNA-miRNA-mRNA ceRNA network was integrated by Cytoscape software, which contained one lncRNA, 79 miRNAs and 13 mRNAs (Figure 2A). From the lncRNA-miRNA-mRNA ceRNA network, two potential regulatory axes (LINC02432/hsa-miR-98–5p/HK2 and LINC02432/hsa-miR-133b/MET) were identified. To select the most meaningful ceRNA regulatory axis in this study, we only selected mRNAs, miRNAs, or lncRNAs that were significantly related to the prognosis of PAA. First, by correlation analysis of each element in the two potential regulatory axes, we found that LINC02432 was positive correlated with HK2 and MET, and was negatively correlated with hsa-miR-98–5p (Figure 2B), but not correlated with hsa-miR-133b (Supplementary Figure S5A). At the same time, hsa-miR-98–5p was significantly negatively correlated with HK2 (Figure 2B), while hsa-miR-133b was not correlated with MET (Supplementary Figure S5B). Furthermore, Prognostic survival analysis showed LINC02432 high expression, hsa-miR-98–5p low expression, and HK2 high expression were correlated with poor prognosis (Figure 2C). As shown in Supplementary Figure S5C, the expression of hsa-miR-133b in pancreatic cancer was not associated with prognosis. Therefore, the LINC02432/hsa-miR-98–5p/HK2 axis is considered to be the most meaningful glycolysis-related ceRNA regulatory axis. Analysis of the Clinical Proteomic Tumor Analysis Consortium (CPTAC) database using UALCAN found that protein expression levels of HK2 were significantly increased in PAAD tissues compared with adjacent healthy tissues (Figure 2D). Moreover, the IHC results from the HPA database showed HK2 was highly expressed in PAAD tissues, and the elevated HK2 was mainly localized in the cytoplasm of PAAD cells (Figure 2E). Cytoplasmic lncRNAs usually acted as ceRNA by binding miRNAs. We found that LINC02432 was expressed in the cytoplasm through the lncLocator website (Figure 2F). A schematic figure describing the LINC02432/hsa-miR-98–5p/HK2 axis showing that LINC02432 regulates glycolytic rate-limiting enzyme HK2 expression and PAAD progression by acting as a ceRNA against hsa-miR-98–5p (Figure 2G). Moreover, the predicted targeting sites of hsa-miR-98–5p on the LINC02432 and HK2 were identified (Figure 2H). We found two putative binding sites for has-miR-98–5p in LINC02432, located at 617–624 bp (Site 1) and 829–836 bp (Site 2).

### 3.3 Construction of the Three-Gene-Based PAAD Prognostic Model

**FIGURE 3:**
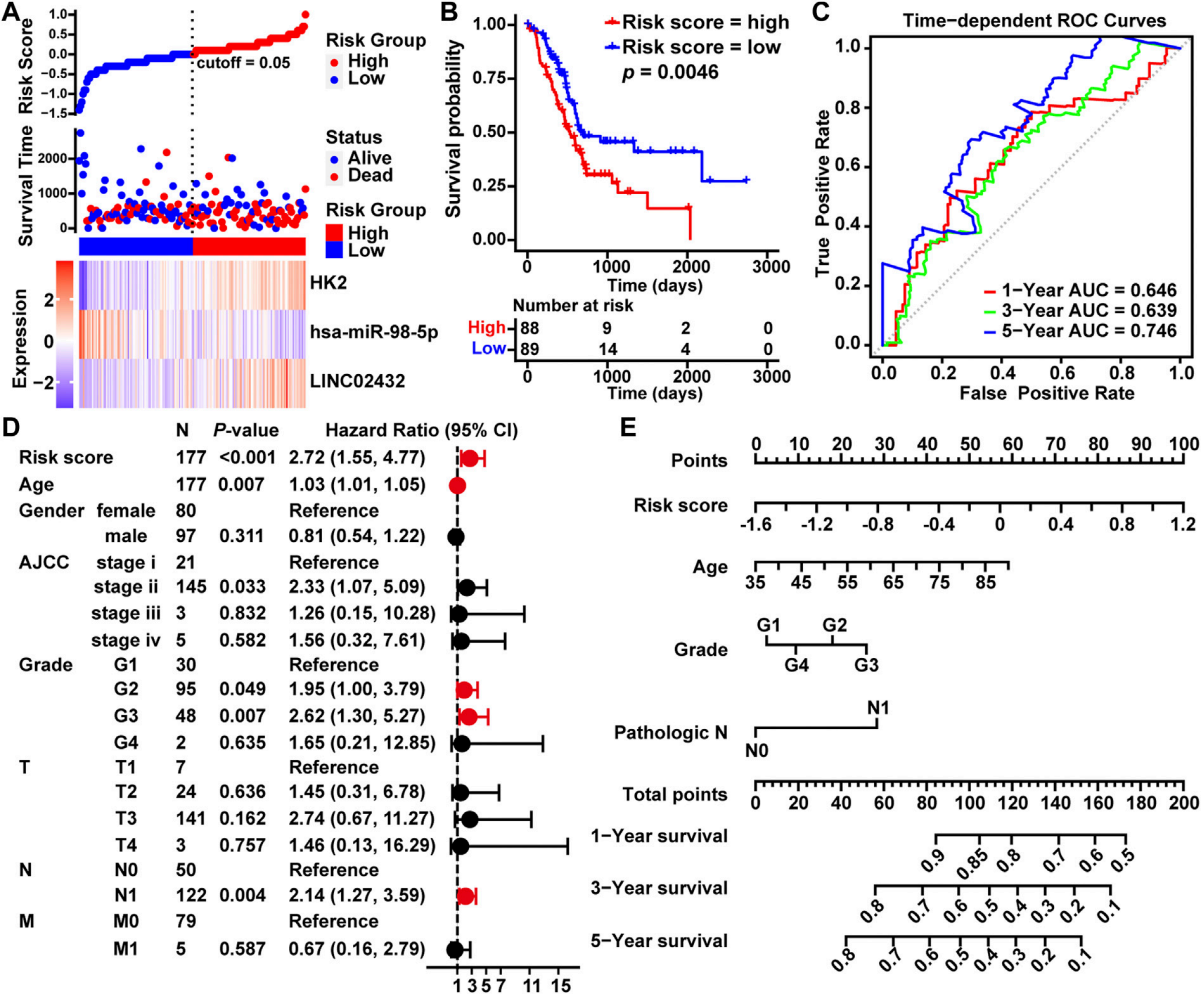
Construction of a prognostic model of PAAD based on the LINC02432/hsa-miR-98–5p/HK2 ceRNA network. **(A)** Risk curves and scatter plots for each sample in the TCGA-PAAD cohort after rearrangement by the ggrisk algorithm. The heat map exhibited the expression levels of LINC02432, hsa-miR-98–5p, and HK2 in the high risk score and low risk score groups. **(B)** Kaplan-Meier analysis showing overall survival in low-risk and high-risk patient groups. **(C)** The time-dependent ROC curves and AUC for 1-year, 3-years, and 5-years overall survival. **(D)** Univariate Cox regression analysis was used to assess the association of clinicopathological features and risk scores. **(E)** A nomogram model was constructed using four independent prognostic factors (risk score, age, grade, and pathological T).

To estimate the association between LINC02432/hsa-miR-98–5p/HK2 axis and clinical prognosis, all 177 TCGA PAAD samples were randomly divided into a training cohort (n = 133) and a testing cohort (n = 44) at a ratio of 3:1. The risk curves, scatterplots and Kaplan-Meier curve analysis were performed in training cohort. The risk curves and scatterplots showed that PAAD patients in the high risk group had higher risk factors and mortality in the training cohort. Heat map showing the expression profiles of the LINC02432/hsa-miR-98–5p/HK2 axis in the training cohort (Supplementary Figure S6A). The result of the Kaplan-Meier curve showed that the overall survival of the high risk group was significantly lower than that of the low risk group in the training cohort (Supplementary Figure S6B). This result was consistent with that of the testing cohort (Supplementary Figures S6C,D) and the entire cohort (Figures 3A,B). In addition, we executed time-dependent ROC curve analysis to assess the sensitivity and specificity of survival prediction for the three-gene signature in TCGA. The AUC values of the risk scores corresponding to 1-year, 3-years, and 5-years survival were 0.646, 0.639, and 0.746, respectively (Figure 3C). This further confirms the high sensitivity and specificity of the three-gene signature as a reliable predictor of overall survival in PAAD. The univariate Cox regression demonstrated that risk score, age, grade, and pathological N could forecast poorer PAAD survival (Figure 3D). As shown in Figure 3E, we created a nomogram model combining the three-gene-based risk score with clinicopathological characteristics (age, grade and pathological N) to evaluate the probability of survival at 1-year, 3-years, and 5-years overall survival in PAAD. Our results demonstrated that the LINC02432/hsa-miR-98–5p/HK2 ceRNA axis might be especially important for the development and prognosis of PAAD by influencing glycolytic activity.

### 3.4 Glycolysis-Related LINC02432/Hsa-miR-98–5p/HK2 Axis Inhibited Ferroptosis

**FIGURE 4:**
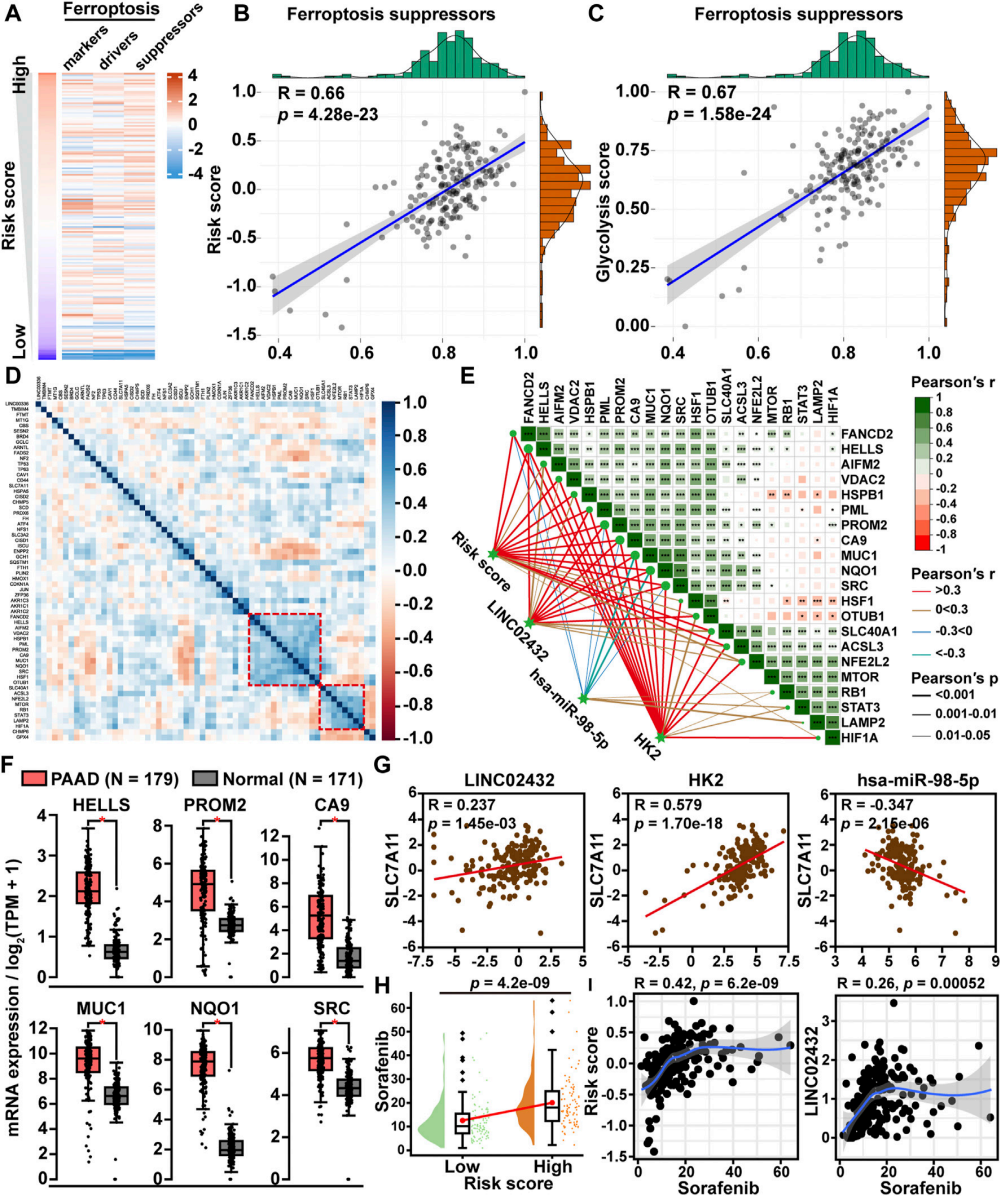
The correlation between LINC02432/hsa-miR-98–5p/HK2 axis and ferroptosis. **(A)** Heatmap of ssGSEA scores for the three ferroptosis-related genes in PAAD samples. **(B)** Dot plot of Pearson correlation between ceRNA risk score and ferroptosis suppressor gene set score. **(C)** The Pearson correlation was used to assess the relationship between glycolysis score and ferroptosis suppressor gene set score. **(D)** Correlation heatmap showing the clustering of ferroptosis suppressor gene expression. **(E)** The correlation heatmap demonstrated the relationship between the LINC02432/hsa-miR-98–5p/HK2 axis and two ferroptosis suppressor gene clusters. **(F)** GEPIA data analysis exhibited the differential expression of HELLS, PROM2, CA9, MUC1, NQO1, and SRC in PAAD compared to normal tissue. **(G)** Pearson correlation analysis was applied to assess the relationship between SLC7A11 and LINC02432/hsa-miR-98–5p/HK2 axis. **(H)** Comparison of sorafenib IC50 between low and high risk score groups in PAAD. **(I)** Correlation between LINC02432 expression and sorafenib IC50 values.

To investigate the role of the glycolysis-related ceRNA network in regulating the ferroptosis pathway, we first downloaded a total of 259 ferroptosis-related genes from the FerrDb database, including 108 driver genes, 69 repressor genes, and 111 marker genes. Then, the ssGSEA scores of the three ferroptosis-related gene sets were plotted through a heatmap in PAAD samples (Figure 4A). We found that the correlation of ceRNA risk score was strongest with the ferroptosis suppressor gene set score. Pearson correlation analysis revealed that ceRNA risk score was significantly positively correlated with ferroptosis suppressor gene sets score (R = 0.66, *p* < 0.001) (Figure 4B). Meanwhile, we found a significant positive correlation between the glycolysis score and the ferroptosis inhibitory genome score (Figure 4C). Following the correlation heatmap cluster analysis of ferroptosis suppressor gene expression data, two clusters of genes were identified as key ferroptosis suppressor genes in PAAD (Figure 4D). Further, we analyzed the correlation between the ceRNA axis and the two clusters of ferroptosis suppressor gene. We found six ferroptosis suppressor genes (HELLS, PROM2, CA9, MUC1, NQO1, and SRC) were significantly positively correlated with risk score, LINC02432, and HK2 and significantly negatively correlated with hsa-miR-98–5p (Figure 4E). GEPIA expression analysis showed that the mRNA expression levels of these six ferroptosis suppressor genes were significantly higher in PAAD tissues than in adjacent normal tissues (Figure 4F). Kaplan-Meier survival analysis demonstrated that high expression of HELLS, PROM2, CA9, MUC1, NQO1, and SRC was significantly associated with a poor prognosis in PAAD patients (Supplementary Figure S7). To gain more evidence that the ceRNA network regulates ferroptosis in pancreatic cancer, we analyzed the relationship between the ceRNA axis and SLC7A11. We found that SLC7A11 expression was positively correlated with the expression of LINC02432 and HK2, and negatively correlated with the expression of hsa-miR-98–5p (Figure 4G). In addition, sorafenib was a ferroptosis inducer. Drug sensitivity analysis showed that the IC50 of sorafenib in the high risk group was significantly higher than that in the low risk group (Figure 4H). Correlation analysis indicated that the expression of LINC02432 was positively correlated with sorafenib IC50 in PAAD (Figure 4I).

### 3.5 CeRNA Risk Score Was Positively Correlated With M0 Macrophages Infiltration

**FIGURE 5:**
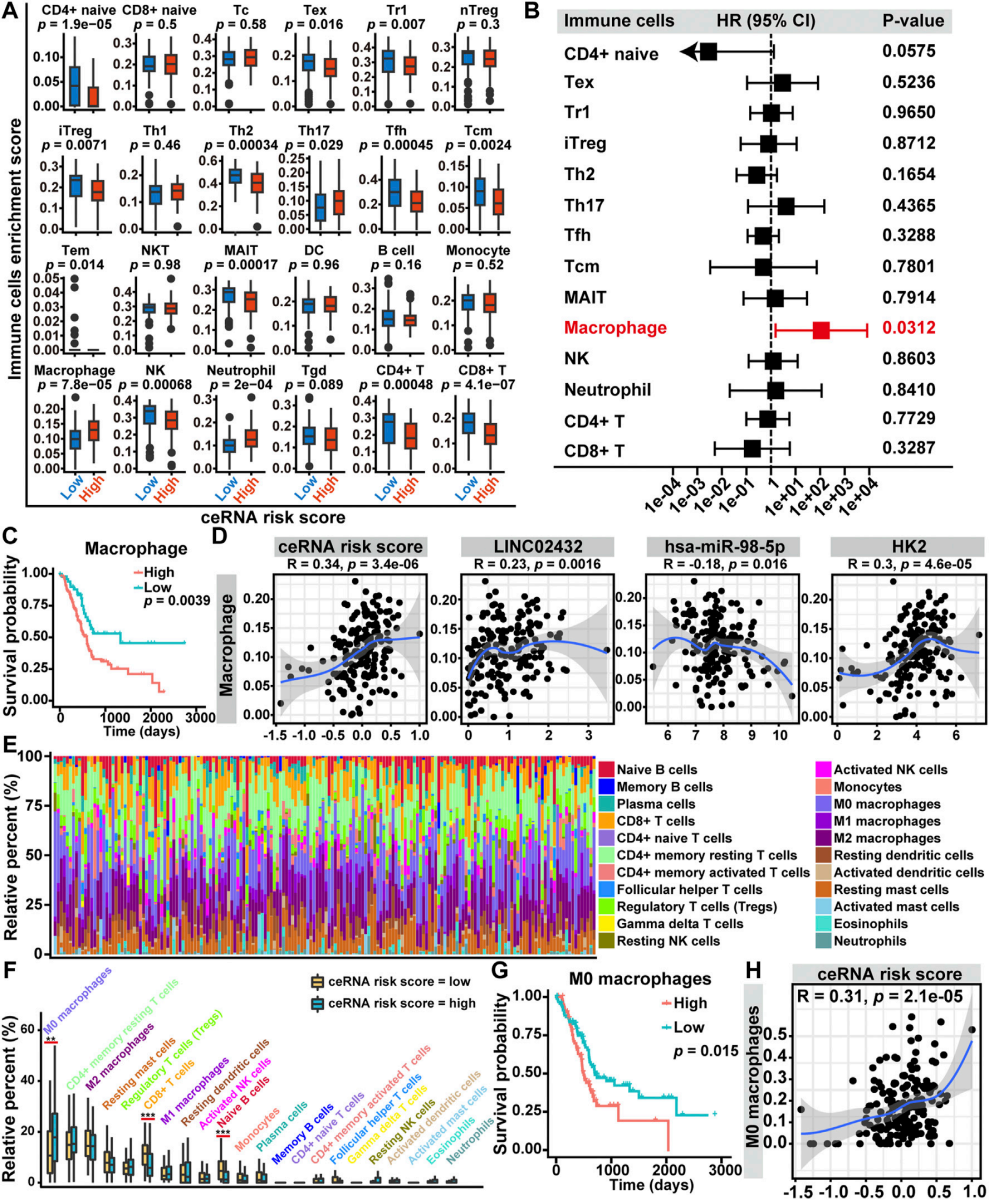
Relationship between LINC02432/hsa-miR-98–5p/HK2 ceRNA network and tumor-infiltrating immune cells in the TME of PAAD. **(A)** The box plots showed the differences in the proportions of 24 tumor-infiltrating immune cells among the high ceRNA risk score and low ceRNA risk score groups. **(B)** Univariate Cox regression analysis of 14 differentially expressed tumor-infiltrating immune cells. **(C)** Kaplan-Meier curve analysis of overall survival in high macrophages fraction and low macrophages fraction groups. **(D)** The Pearson correlation among the fraction of macrophages and the LINC02432/hsa-miR-98–5p/HK2 ceRNA network. **(E)** Based on the CIBERSORT algorithm, histograms showed the infiltration of 22 immune cells in each sample. **(F)** The box plots showed the difference of the 22 infiltrating immune cells between groups with different ceRNA risk scores. **(G)** Kaplan-Meier curves of M0 macrophages for overall survival in PAAD patients. **(H)** The Pearson correlation between the fraction of M0 macrophages and the ceRNA risk score.

To assess the relationship among ceRNA risk scores and immune cell infiltration, we applied the ImmuCellAI tool to calculate the abundance of 24 immune cell subsets in PAAD samples. As shown in Supplementary Figure S8A, the association among immune cell infiltration and ceRNA risk score was demonstrated by a heat map. We then investigated the infiltration differences of 24 immune cells between the high ceRNA risk score and low ceRNA risk score groups. We discovered that the expression signatures for three types of infiltrating cells (Th17 cells, macrophages, and neutrophils) were elevated in the high ceRNA risk score group. The low ceRNA risk score group had higher infiltration levels of CD4^+^ naive cells, Tex cells, Tr1 cells, iTreg cells, Th2 cells, Tfh cells, Tcm cells, MAIT cells, NK cells, CD4^+^ T cells and CD8^+^ T cells (Figure 5A). Univariate Cox regression analysis revealed that, among the 14 differentially infiltrating immune cells, only macrophages was a significant prognostic factor for the overall survival of PAAD patients (Figure 5B). Furthermore, Kaplan-Meier curve analysis suggested that high macrophage infiltration PAAD patients had poor overall survival (Figure 5C). Finally, the Pearson correlation analysis revealed that macrophage infiltration levels were positively correlated with ceRNA risk score, LINC02432, and HK2, which was also negatively correlated with hsa-miR-98–5p (Figure 5D).

The macrophages could be split into three subtypes (M0, M1, and M2), of which M0 was the inactive subtype and could differentiate into either the M1 or M2 activated subtypes. In the present study, we investigated the correlations among ceRNA risk scores and three macrophage subtypes by the CIBERSORT algorithm. The histogram of immune cell infiltration clearly showed that PAAD patients had a high abundance of M0 macrophages, M2 macrophages, and CD4^+^ memory resting T cells (Figure 5E). In addition, the box plot of the discrepancy of immune cell infiltration revealed that the abundance of CD8^+^ T cells and naive B cells were significantly higher in the low risk group, and the levels of M0 macrophages was significantly higher in the high risk group (Figure 5F). Subsequently, we performed a Kaplan-Meier survival analysis of the three differentially infiltrating immune cells. We found that only higher M0 macrophage infiltration was significantly associated with decreased overall survival (Figure 5G). However, more CD8^+^ T cells and naive B cells infiltration were not significantly associated with overall survival (Supplementary Figure S8B). Then, a Pearson correlation analysis was performed to assess the correlation between M0 macrophages infiltration and ceRNA risk score. We discovered that the infiltration level of M0 macrophages was positively correlated with ceRNA risk score (Figure 5H).

### 3.6 CeRNA Risk Scores Were Associated With Chemokine Levels in PAAD

**FIGURE 6:**
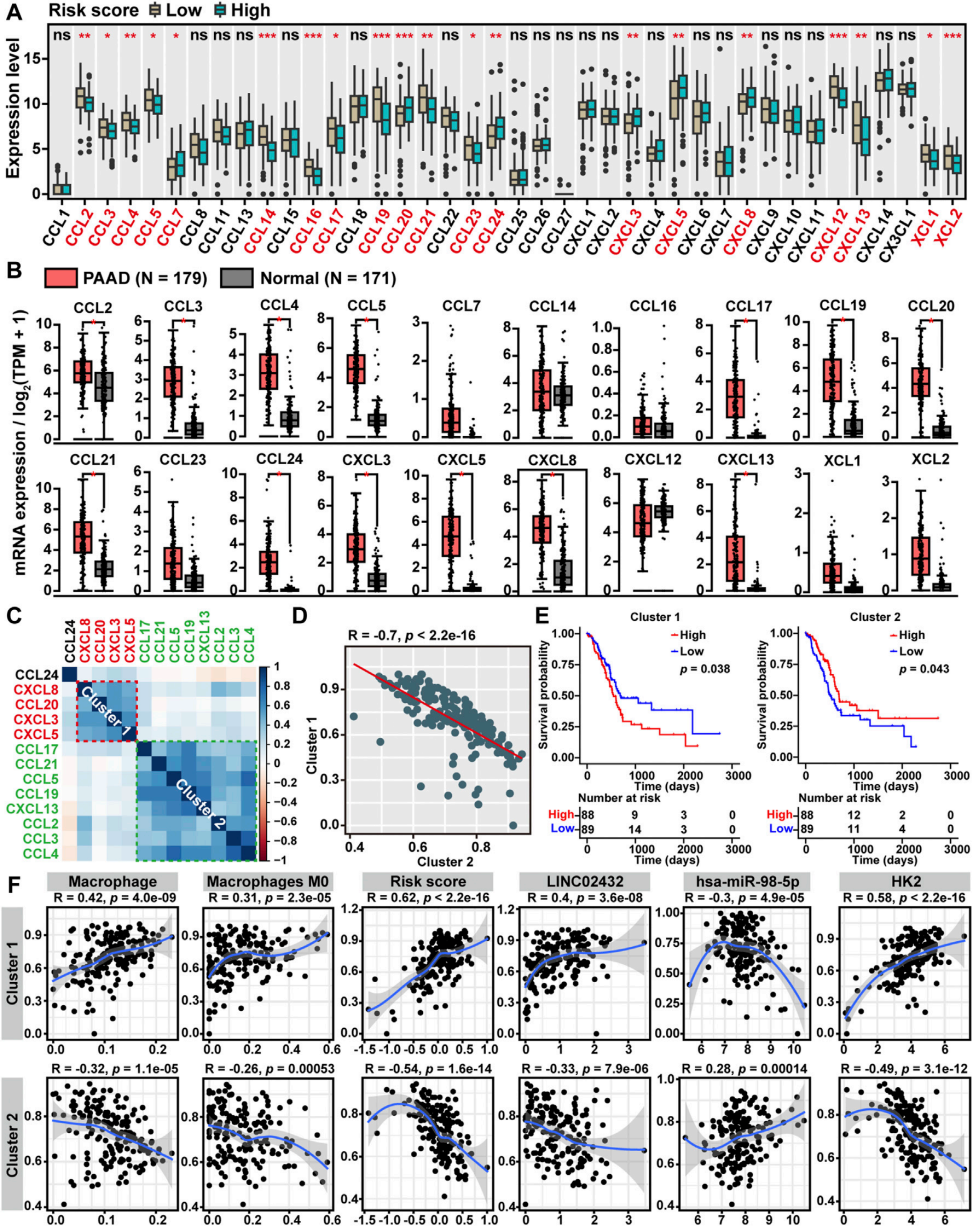
Correlation between ceRNA risk scores and chemokine levels. **(A)** The box plots showed the expression differences of 40 chemokines among high risk score and low risk score groups of TCGA-PAAD patients. **(B)** The box plot revealed the expression levels of chemokines between PAAD tissues and normal tissues by the GEPIA2 tool. **(C)** The clustered heatmap showed the Pearson correlation of 13 differentially expressed chemokines. **(D)** The Pearson correlation analysis of two chemokine clusters based on the ssGSEA scores. **(E)** The Kaplan-Meier curves of PAAD overall survival based on the ssGSEA scores of two chemokine clusters. **(F)** The Pearson correlation analysis of two chemokine clusters with LINC02432/hsa-miR-98–5p/HK2 ceRNA network and macrophages infiltration levels.

Chemokines, also referred to as chemotactic cytokines, have long been recognized as critical mediators of the inflammatory response and played a key role in the infiltration and activation of immune cells. To elucidate the relationship between ceRNA risk scores and chemokine levels in PAAD, we compared the gene expression levels of 40 known chemokines among the high-risk score and low-risk score groups. We found that six chemokines (CCL7, CCL20, CCL24, CXCL3, CXCL5, and CXCL8) were upregulated in the high-risk score group, while 14 chemokines (CCL2, CCL3, CCL4, CCL5, CCL14, CCL16, CCL17, CCL19, CCL21, CCL23, CXCL12, CXCL13, XCL1 and XCL2) were downregulated in the high risk score group (Figure 6A). As showed in Figure 6B, the GEPIA analysis showed that 13 of the above 20 chemokines were significantly upregulated in PAAD. Furthermore, through the cluster heatmap of Pearson correlation hierarchical clustering analysis based on the 13 differentially expressed chemokines pattern, we identified two gene clusters: cluster one included four genes (CXCL3, CXCL5, CXCL8, and CCL20), and cluster two included eight genes (CCL2, CCL3, CCL4, CCL5, CCL17, CCL19, CCL21, and CXCL13) (Figure 6C). The Pearson correlation analysis demonstrated that ssGSEA score of cluster one was negatively correlated with cluster two score (Figure 6D). Moreover, Kaplan-Meier survival analysis showed that high cluster one score patients had worse overall survival, and high cluster two score patients had better overall survival (Figure 6E). Finally, we performed Pearson correlation analysis to investigate the relationship between LINC02432/hsa-miR-98–5p/HK2 ceRNA network and two chemokine clusters. As shown in Figure 6F, we found that cluster one score was positively correlated with macrophages, M0 macrophages, risk score, LINC02432, and HK2, and negatively correlated with hsa-miR-98–5p. On the contrary, cluster two score was negatively correlated with macrophages, M0 macrophages, risk score, LINC02432, and HK2, as well as positively correlated with hsa-miR-98–5p.

### 3.7 Correlation Between ceRNA Risk Scores and Immune Checkpoint Genes

**FIGURE 7:**
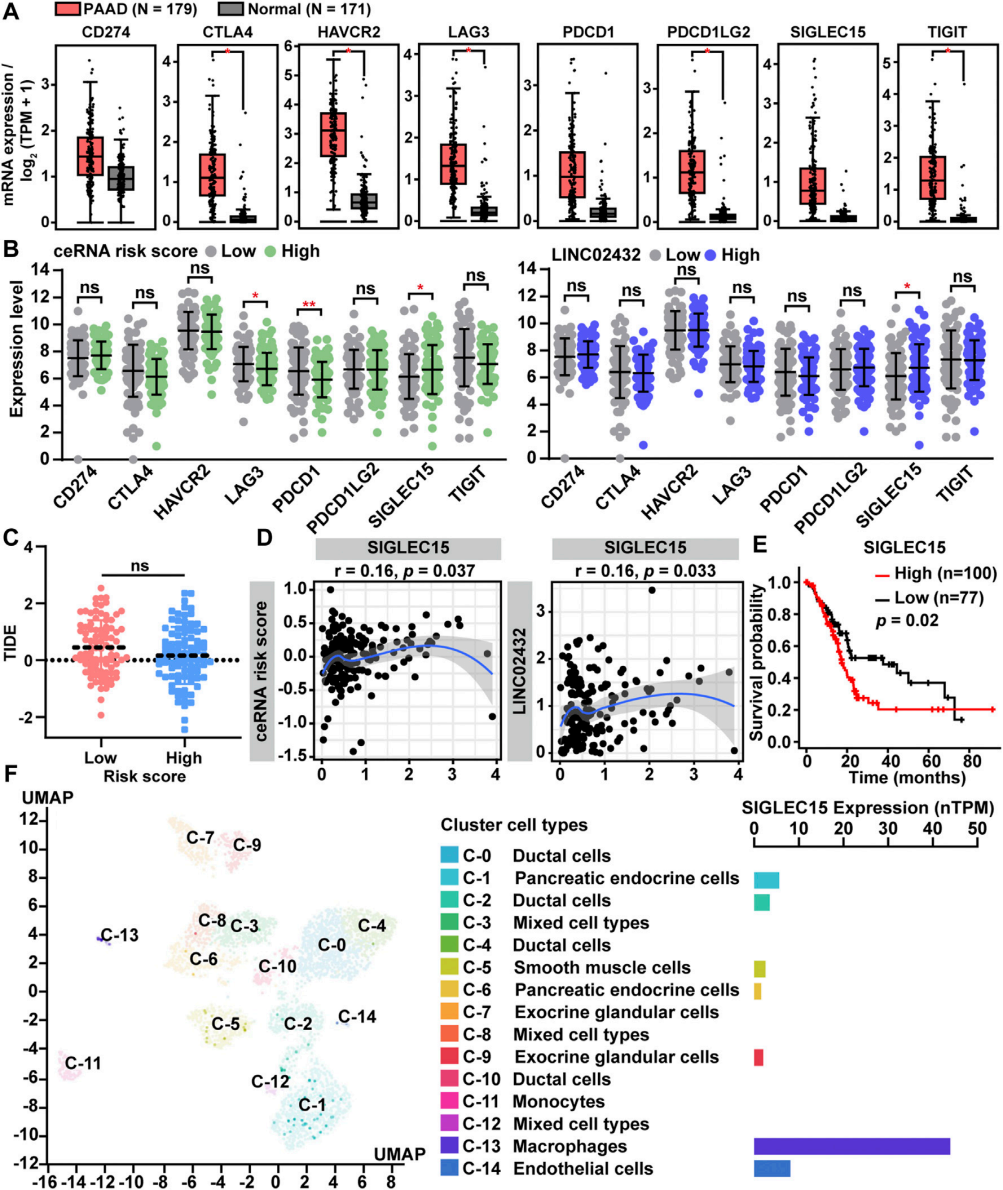
Correlation of ceRNA risk score with the expression of immune checkpoint genes for PAAD. **(A)** The expression of eight immune checkpoint genes between PAAD tissues and normal pancreas tissues. **(B)** The expression differences of eight immune checkpoint genes among different groups. **(C)** Statistical analysis of TIDE scores between low and high risk groups. **(D)** The correlation between SIGLEC15 expression and ceRNA risk score was analyzed by Pearson correlation analysis. **(E)** The prognostic significance of SIGLEC15 assessed by Kaplan-Meier analysis. **(F)** SIGLEC15 expression was identified in different human pancreas cell types by scRNA-seq data from the HPA.

Immune checkpoint genes, as key genes regulating immune responses, were frequently dysregulated in tumor and immune cells, leading to immune evasion of cancer cells. In order to further analyze the association between ceRNA risk scores and immune checkpoint genes, we first compared differential expression of the eight immune checkpoint genes (CD274, CTLA4, HAVCR2, LAG3, PDCD1, PDCD1LG2, SIGLEC15, and TIGIT) in PAAD samples and normal samples. We found that all eight immune checkpoint genes had high expression levels in PAAD tissues (Figure 7A). As presented in Figure 7B, LAG3 and PDCD1 were downregulated in the high risk group, but they were not significantly different between LINC02432 high and low expression groups. Only SIGLEC15 was significantly up-regulated in the high-risk score group and the high LINC02432 expression group. TIDE analysis showed no correlation between ceRNA risk score and immunotherapy (anti-PDCD1 therapy and anti-CTLA4 therapy) (Figure 7C). We then assessed the correlation between SIGLEC15 and ceRNA risk score by Pearson correlation analysis. We found that SIGLEC15 expression was significantly positively correlated with ceRNA risk score and LINC02432 expression (Figure 7D). Moreover, Kaplan-Meier analysis indicated that high expression of SIGLEC15 could predict poor prognosis of PAAD patients (Figure 7E). This was uniform with the prognostic results of LINC02432 and HK2. To further validate the expression of SIGLEC15 across different cell types in pancreas tissues, we used the scRNA-seq data from the HPA. We discovered that SIGLEC15 was expressed almost exclusively in cluster c-13 corresponding to macrophages of the pancreas (Figure 7F).

### 3.8 LINC02432/Hsa-miR-98–5p/HK2 Axis Was Related to TMB in PAAD

**FIGURE 8:**
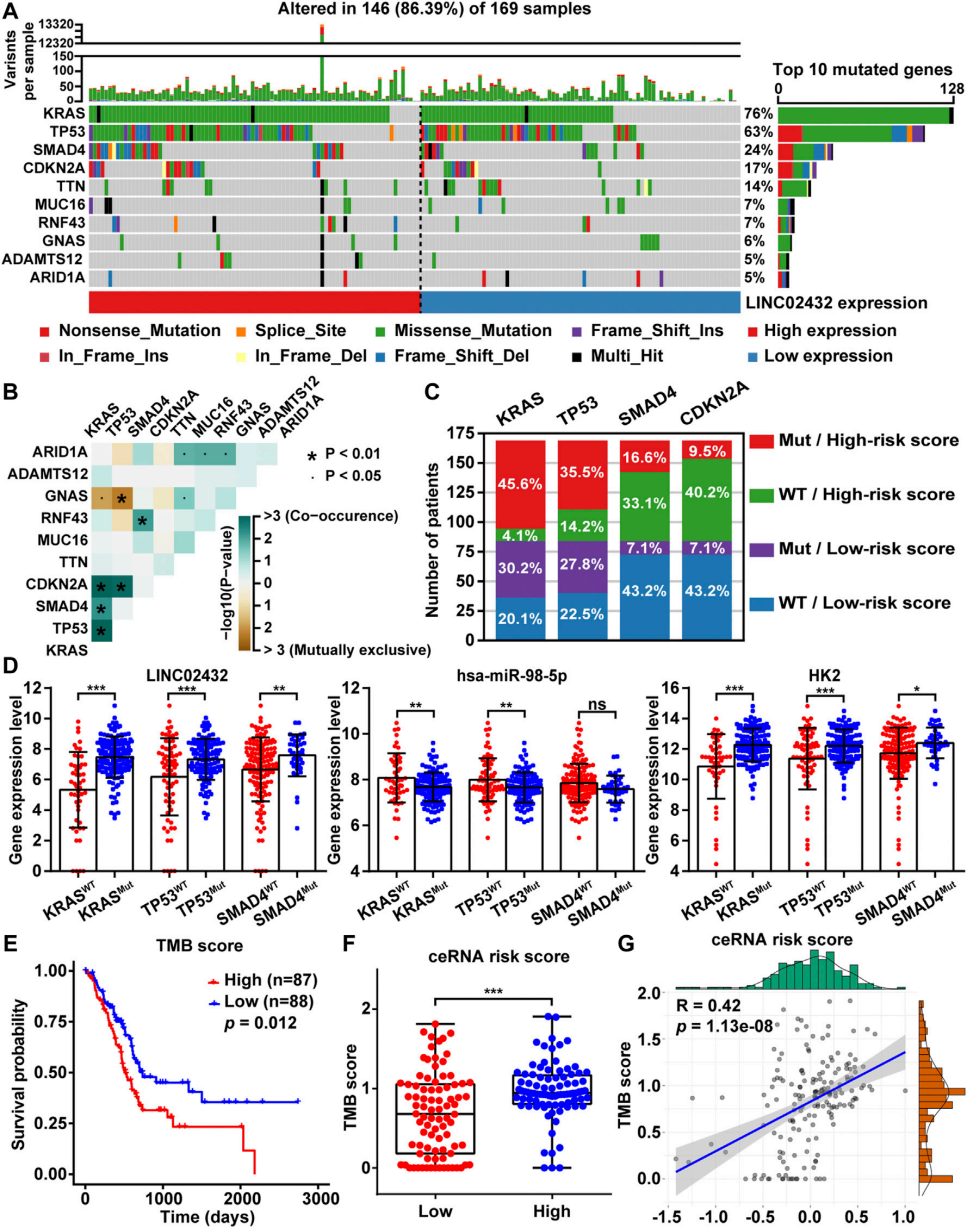
The relationship between TMB and risk score. **(A)** The mutation signatures of the top 10 significant mutated genes in the high and low LINC02432 expression groups. **(B)** The heatmap showing the co-occurrence and mutually exclusive mutations within the top 10 frequently mutated genes. **(C)** The distribution ratio of PAAD patients in different risk subgroups. **(D)** The expression levels of LINC02432, hsa-miR-98–5p, and HK2 in the WT and Mut groups of KRAS, TP53, and SMAD4 genes. **(E)** Kaplan-Meier survival analysis for PAAD patients stratified by TMB score. **(F)** TMB score between patients from the high and low ceRNA risk score subgroups. **(G)** The Pearson correlation analysis between risk score and TMB score.

TMB, the total number of somatic mutations in tumors, was emerging as a promising biomarker for immunotherapy response in cancer patients. To analyze the relationship between ceRNA risk scores and TMB, somatic mutation analysis was performed to show the top 10 frequently mutated genes associated with PAAD tumorigenesis. The mutation frequency of high LINC02432 expression group was mostly higher than that of low LINC02432 expression group, especially KRAS, TP53, SMAD4 and CDKN2A (Figure 8A). Subsequently, the results of mutual exclusion and co-occurrence analysis of the top 10 frequently mutated genes were shown in Figure 8B. KRAS mutations, as the highest frequency mutations in PAAD, were co-occurrence with TP53, SMAD4, and CDKN2A mutations. The 169 PAAD samples were divided into four groups according to gene mutational status (WT: wild type; MUT: mutant) and high/low risk score. As shown in Figure 8C, the mutation frequencies of KRAS, TP53, and SMAD4 genes were significantly higher in the high-risk score patients than that in the low-risk score patients. Furthermore, we analyzed the differential expression of the LINC02432/hsa-miR-98–5p/HK2 ceRNA axis in the MUT and WT groups. The results demonstrated that LINC02432 and HK2 were significantly upregulated in the KRAS, TP53, and SMAD4 mutant groups, and hsa-miR-98–5p was significantly downregulated in the KRAS and TP53 mutant groups (Figure 8D). Based on TCGA mutation data of whole-exome sequencing, we calculated the TMB score for each PAAD patient. The survival curve showed that the survival time of patients in the high TMB score group was significantly shorter than that in the low TMB score group (Figure 8E). Meanwhile, patients with high ceRNA risk score had remarkably higher TMB score than patients with low ceRNA risk score (Figure 8F). Finally, Pearson correlation analysis indicated that TMB scores were positively correlated with the ceRNA risk score in PAAD patients (Figure 8G). Previous studies reported that patients with high TMB had better response to immunotherapy. Combined with the above results, PAAD patients in the high risk group might be more sensitive to anti-SIGLEC15 immunotherapy, but not to anti-PD-L1 and anti-CTLA4 immunotherapy.

### 3.9 Correlation Analysis Between Risk Score and the Drug Sensitivity

**FIGURE 9:**
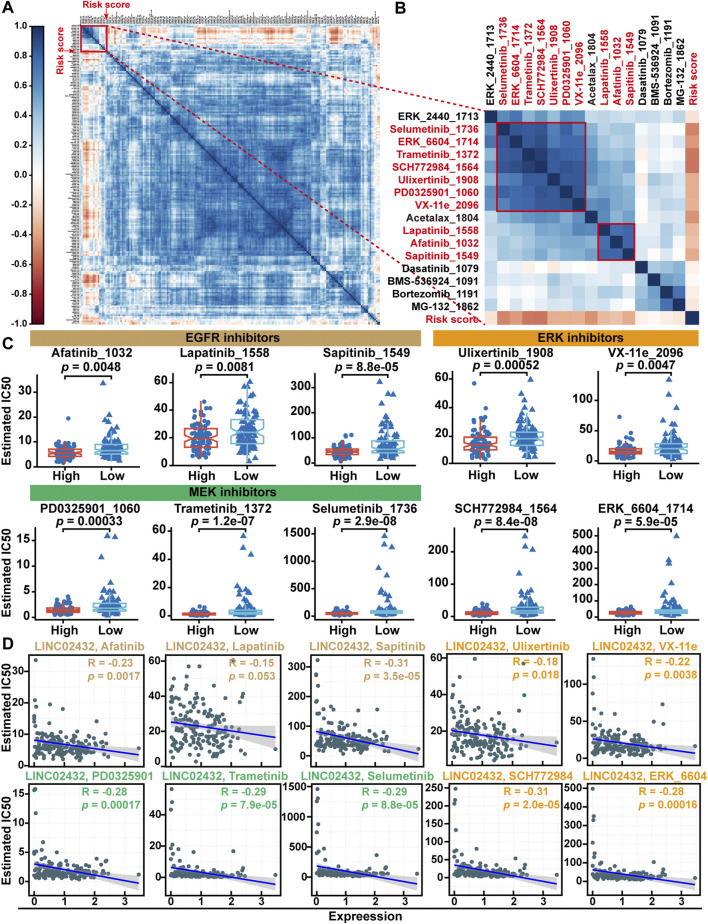
Drug sensitivity analysis of the risk score. **(A)** The correlation heatmap between the IC50 values of anti-tumor drugs and risk score. **(B)** Correlation heatmap showing the association of two clusters drugs (containing 10 compounds) with risk scores. **(C)** Differential analysis of drug IC50 values between high risk score and low risk score groups. **(D)** Scatter plots showed correlation between IC50 values of 10 compounds and lINC02432 expression.

Previous studies have shown that the high rates of tumor cell glycolysis would make them resistant to many forms of chemotherapy. To identify potential drugs for PAAD patients with high risk scores, we first estimated IC50s for 198 drugs using the oncoPredict package in R software. As shown in the correlation heatmap, the results showed that the risk score was positively correlated with the IC50 values of most anti-tumor drugs (Figure 9A). This suggests that patients with high-risk had less sensitive to most antitumor drugs. Further analysis found that only two clusters containing 10 compounds were negatively associated with risk scores (Figure 9B). Meanwhile, differential analysis of drug IC50 values showed that EGFR inhibitors (afatinib, lapatinib and sapitinib), MEK inhibitors (PD0325901, trametinib and selumetinib) and ERK inhibitors (Ulicocitinib, VX-11e, SCH772984, and ERK_6604) had lower IC50 values in the high risk group compared with the low risk group (Figure 9C). Pearson correlation analysis indicated that the IC50 of the other nine drugs except lapatinib were significantly positively correlated with lINC02432 expression (Figure 9D). These results suggested that high-risk PAAD patients with high immune infiltration and TMB might be more sensitive to EGFR, MEK, and ERK inhibitors.

## 4 Discussion

Cancer cells almost universally showed metabolic reprogramming with an increased reliance on aerobic glycolysis (Warburg effect) (Telarovic et al., 2021). Recent studies indicated that metabolically reprogrammed aerobic glycolysis promoted cell proliferation and cell metastasis of PAAD (An et al., 2017; Fan et al., 2019; Cao et al., 2020; Yu et al., 2021). In PAAD, targeting aerobic glycolysis is considered a potential therapeutic strategy to improve patient outcomes (Zhang and Zhang, 2021). Although there is a lot of research on the involvement of glycolysis in PAAD progression, there were seldom reports on studies involving glycolysis-related biomarkers and prognosis in PAAD patients. LncRNA is an RNA transcript that does not encode a protein and is longer than 200 nucleotides. Mounting evidence has shown that lncRNAs, functioning as a ceRNA by competitively binding to miRNAs, affect aerobic glycolysis and participate in cancer progression and treatment resistance. For example, Jia et al. demonstrated that the lncRNA LNCAROD induced pyruvate kinase isoenzyme M2 (PKM2) upregulation via sponging miR-145–5p, increased aerobic glycolysis in hepatocellular carcinoma cells, and was eventually involved in tumor malignancy and chemoresistance (Jia et al., 2021). Xu et al. had reported that LINC01448 promoted cell proliferation, cell invasion, and glucose consumption by modulating the miR-505/HK2 pathway in PAAD (Xu Z. et al., 2020). Therefore, in-depth investigation of the mechanism of lncRNA regulation of aerobic glycolysis in PAAD may provide new strategies for clinical tumor management.

In this study, combined with the TCGA and GEO databases, we used bioinformatics technology to obtain the LINC02432/hsa-miR-98–5p/HK2 ceRNA axis, which might influence the development and prognosis of PAAD by affecting glycolytic activity. Current research showed that HK2 and PKM2 were two important enzymes that directly modulate glycolysis. In PAAD, upregulated expressions of HK2 and PKM2 were associated with lactate production and poor clinical prognosis (Bernier et al., 2017; Yu et al., 2021). Numerous studies have shown that hsa-miR-98–5p was downregulated in some malignancies and could function as a tumor suppressor (Jiang F. et al., 2019). Fu et al. suggested that hsa-miR-98–5p inhibited cell proliferation and cell metastasis by downregulating the counter-regulatory mitogen-activated protein four kinase 4 (MAP4K4) in PAAD (Fu et al., 2018). Zhu et al. found that hsa-miR-98–5p inhibited colon cancer cells glycolysis by directly targeting HK2 (Zhu et al., 2017). Additionally, hsa-miR-98–5p has been proved to be a target of lncRNA TMPO-AS1, inhibiting the progression of colorectal cancer cells by downregulating the expression of branched chain amino acid transaminase 1 (BCAT1) (Ye et al., 2022). For the first time, the present study found that hsa-miR-98–5p was sponged by the LINC02432 and regulated HK2 in PAAD cells. To our knowledge, LINC02432 had only been reported to be highly expressed in the kidney and pancreas (Fagerberg et al., 2014; Qu et al., 2021). There was no report about the function of LINC02432. Our research results provided a new potential target for glycolysis-targeted therapy of PAAD.

In the present study, we found that six ferroptosis suppressors (HELLS, PROM2, CA9, MUC1, NQO1, and SRC) were significantly positively associated with the ceRNA network. Jiang et al. found that HELLS interacted with WDR76 (WD repeat domain 76) to inhibit ferroptosis by activating metabolic genes, including glucose transporter 1 (GLUT1), and sterol-CoA desaturase 1 (SCD1), and fatty acid desaturase 2 (FADS2) (Jiang et al., 2017; Mazhar et al., 2021). Luo et al. revealed that PROM2 promoted iron export and ferroptosis resistance *via* formation of multivesicular bodies (MVBs) in BLCA (Luo et al., 2021). Li et al. demonstrated that carbonic anhydrase 9 (CA9), a classical HIF1A target gene, promoted malignant mesothelioma resistance to ferroptosis and apoptosis under hypoxia (Li et al., 2019; Tang et al., 2021). Meanwhile, MUCIN 1 (MUC1) could bind to the CD44 variant to enhance the stability of SLC7A11, thereby inhibiting erastin-induced ferroptosis in triple-negative breast cancer cells (Hasegawa et al., 2016; Tang et al., 2021). NAD(P)H: ubiquinone oxidoreductase-1 (NQO1) functioned as a CoQ oxidoreductase and mitochondrial ROS inhibitor and has been demonstrated to suppress ferroptosis (Sun et al., 2016; Peng et al., 2022). Studies have shown that the activation of SRC proto-oncogene non-receptor tyrosine kinase (SRC) can inhibit cancer cell ferroptosis by inhibiting the expression of acyl-CoA synthetase long-chain family member 4 (ACSL4) (Brown et al., 2017; Ye et al., 2021). This further suggested the regulatory role of LINC02432/hsa-miR-98–5p/HK2 axis on ferroptosis inhibition. At the same time, studies have confirmed that SLC7A11 is a key regulator of ferroptosis in response to sorafenib (Huang et al., 2021). Sorafenib could induce ferroptosis by the inhibition of SLC7A11 (glutamate-cystine exchanger xCT) (Wang H. et al., 2021; Wang et al., 2022). In this study, we found that the expression of SLC7A11 was positively correlated with LINC02432 and HK2. This may be a possible mechanism that the patients in the high-risk group were more insensitive to sorafenib.

Pancreatic TME, and in particular infiltrating inflammatory cells (largely macrophages), represented an important contributing factor to PAAD aggressiveness and resistance to treatment (Mohseni et al., 2021). Macrophages in the TME were often called tumor-associated macrophages and contained three phenotypes: M0, M1, and M2 (Xu C. et al., 2020). Studies have shown that M1 macrophages had pro-inflammatory and anti-tumor effects and were associated with good prognosis in certain cancers. M2 macrophages had immunosuppressive and tumor-promoting effects (Zhang J. et al., 2020; Yu et al., 2020). M0 macrophages, as a non-polarized subtype, were independent predictors of poor prognosis in PAAD patients (Zhang J. et al., 2020; Xu C. et al., 2020). Tekin et al. discovered that M0 macrophages secreted matrix metalloprotease 9 (MMP9) to induce mesenchymal transition in PAAD cells via protease-activated receptor 1 (PAR1) activation (Tekin et al., 2020). Consequently, we explored the relationship among ceRNA network and tumor-associated macrophages in PAAD using ImmuCellAI and CIBERSORT algorithms. In this study, we found that LINC02432/hsa-miR-98–5p/HK2 ceRNA risk score was remarkably positively associated with the infiltration level of M0 macrophages. These results strongly suggested that the LINC02432/hsa-miR-98–5p/HK2 axis played an outstanding role in the regulation of PAAD immune cell infiltration.

Chemokines (chemotactic cytokines), as a type of small molecular proteins, have been shown to accelerate cell proliferation, cell invasion, and cell migration, and regulate immune cell infiltration in various tumors (Nagarsheth et al., 2017; Wu and Chu, 2021). Therefore, we elucidated the relationship among ceRNA risk scores and 40 known chemokines levels in PAAD. Found that the LINC02432/hsa-miR-98–5p/HK2 ceRNA risk score was positively correlated with CXCL3, CXCL5, CXCL8 and CCL20. Current research has shown that CXCL3, CXCL5, and CXCL8 were CXC chemokines strongly associated with tumor angiogenesis. CXCL5 was upregulated in PAAD tissues and was associated with poor patients prognosis (Zhang R. et al., 2020). Meanwhile, studies have shown that the GABRP-KCNN4 complex could promote the transcription of CXCL5 and CCL20 by activating NF-kappaB, ultimately inducing macrophage infiltration in PAAD (Jiang S. H. et al., 2019). It suggested that the LINC02432/hsa-miR-98–5p/HK2 axis might be involved in the angiogenesis of PAAD. In addition, we also found ceRNA risk score was significantly negatively correlated with CCL2, CCL3, CCL4, CCL5, CCL17, CCL19, CCL21 and CXCL13. Numerous studies have suggested that CCL2 and CCL5 could promote M2 macrophages generation in TME (Smida et al., 2020; Kang et al., 2021). M2 macrophages secreted chemokines such as CCL17 and CXCL13 (Liu Y. et al., 2020; Xie et al., 2021). Meanwhile, CCL19 and CCL21 were shown to be associated with M1 macrophage chemotaxis (Xuan et al., 2015; Boibessot et al., 2021). Chemokines such as CCL3 and CCL4 were upregulated in M1 macrophages compared to M2 macrophages (Hörhold et al., 2020). These chemokines were lowly expressed in the high risk score group of PAAD patients, which might be the main reason why tumor-associated macrophages maintained an undifferentiated M0 phenotype.

Immune checkpoint blockade has demonstrated substantial usefulness in non-small cell lung cancer, melanoma, renal cancer, and other cancers, while its role in PAAD was limited (Wang G. et al., 2021). TAMs have been found to play a significant function in regulating PAAD tumorigenesis and immune checkpoint responses (Li et al., 2018). Therefore, analyzing the relationship between the macrophages infiltration related ceRNA network and immune checkpoint genes had important guiding significance for the immunotherapy of PAAD. In this study, we found that the transcript levels of eight immune checkpoint genes (CD274, CTLA4, HAVCR2, LAG3, PDCD1, PDCD1LG2, SIGLEC15, and TIGIT) were all upregulated in PAAD tissues. However, only SIGLEC15 were significantly positive correlated with LINC02432/hsa-miR-98–5p/HK2 ceRNA risk score. This might be the reason why the ceRNA risk score was not related to the TIDE score (predictor for anti-PDCD1 and anti-CTLA4 therapy). SIGLEC15 was recently reported as an immunosuppressive molecule expressed by TAMs and upregulated in some solid tumors including PAAD (Li Q. T. et al., 2020; Läubli and Varki, 2020). In the TME, SIGLEC 15 could bind to putative responder protein expressed on CD8^+^ T cells to induce subsequent suppression of antitumor immune responses (Cao et al., 2019). A human Phase I clinical trial is currently underway to evaluate the efficacy of a humanized mAb (NC318) against SIGLEC15 in solid tumors (Wang et al., 2019). So, LINC02432/hsa-miR-98–5p/HK2 axis was therefore suggested as an auxiliary marker for SIGLEC15 blocking immunotherapy, and as a potential therapeutic target for PAAD.

Repeated somatic mutations in specific genes have been identified as potential cancer promoters (Balmain, 2020; Pan et al., 2022). In pancreatic cancer, four genes are often mutated: KRAS, CDKN2A, SMAD4, and TP53 (Ciernikova et al., 2020). Previous studies have shown that KRAS mutations first drived pancreatic precancerous lesions, followed by inactivation of CDKN2A, TP53, and SMAD4 (Kato et al., 2016; Qin et al., 2020). Recent studies have revealed that inactivation of tumor suppressors could promote cellular aerobic glycolysis. For example, PAAD driver (KRAS and TP53 genes) mutations could elevate the expression of glucose transporter 1 (GLUT1), hexokinase 1 (HK1), hexokinase 2 (HK2), and lactate dehydrogenase A (LDHA) (Oba et al., 2018; Chisari et al., 2021). Meanwhile, SMAD4 inactivation in PAAD could promote upregulated expression of PGK1 and enhance glycolysis and tumor invasiveness (Liang et al., 2020; Zhu et al., 2021). Therefore, we analyzed the correlation between LINC02432/hsa-miR-98–5p/HK2 ceRNA network and somatic mutations in PAAD. We found that the ceRNA network was mainly associated with KRAS and TP53 mutations. PAAD patients with KRAS and TP53 mutant genes had high expression levels of LINC02432 and HK2, and low hsa-miR-98–5p expression levels. The number of somatic mutations present in the tumor genome was represented by TMB. High TMB score was associated with the poor prognosis of PAAD patients (Li L. et al., 2021). Meanwhile, high TMB could increase the emergence of neoantigens, thereby enhancing immunotherapy response (Lin et al., 2021). In this study, we found ceRNA risk score was positively associated with TMB score. Our results suggested that PAAD patients with high risk score might be more sensitive to anti-SIGLEC15 immunotherapy. In recent years, the discovery of antitumor targets has led to the development of cancer therapy from traditional cytotoxic drugs to new specific antitumor drugs (Li C. et al., 2020; Li C. et al., 2021). Our drug susceptibility analysis showed that high-risk PAAD patients might be more sensitive to EGFR, MEK, and ERK inhibitors. The results showed that the risk score model based on the LINC02432/hsa-miR-98–5p/HK2 ceRNA network could well predict the drug sensitivity of PAAD patients and guide the clinical selection of appropriate drugs to a certain extent.

## Conclusion

In conclusion, through integrated bioinformatics analysis, we constructed a novel glycolysis-related LINC02432/hsa-miR-98–5p/HK2 ceRNA network in which all RNAs had significant predictive values for PAAD prognosis. At the same time, the ceRNA network was markedly associated with ferroptosis, immune infiltration, tumor mutational burden, and drug sensitivity. The results of the present study may further elucidate the mechanisms underlying PAAD progression and provide novel targets for the treatment of PAAD.

## Data Availability

The datasets presented in this study can be found in online repositories. The names of the repository/repositories and accession number(s) can be found in the article/Supplementary Material.
