# Supplementary material for: Glycolysis-Related LINC02432/Hsa-miR-98–5p/HK2 Axis Inhibits Ferroptosis and Predicts Immune Infiltration, Tumor Mutation Burden, and Drug Sensitivity in Pancreatic Adenocarcinoma
Source: Front Pharmacol. 2022 Jun 20;13:937413. doi: 10.3389/fphar.2022.937413 (PMC9251347; doi:10.3389/fphar.2022.937413)
Supplement: Supplementary file 1 [file DataSheet1.DOCX]

Supplementary Material

# Supplementary Figures

**
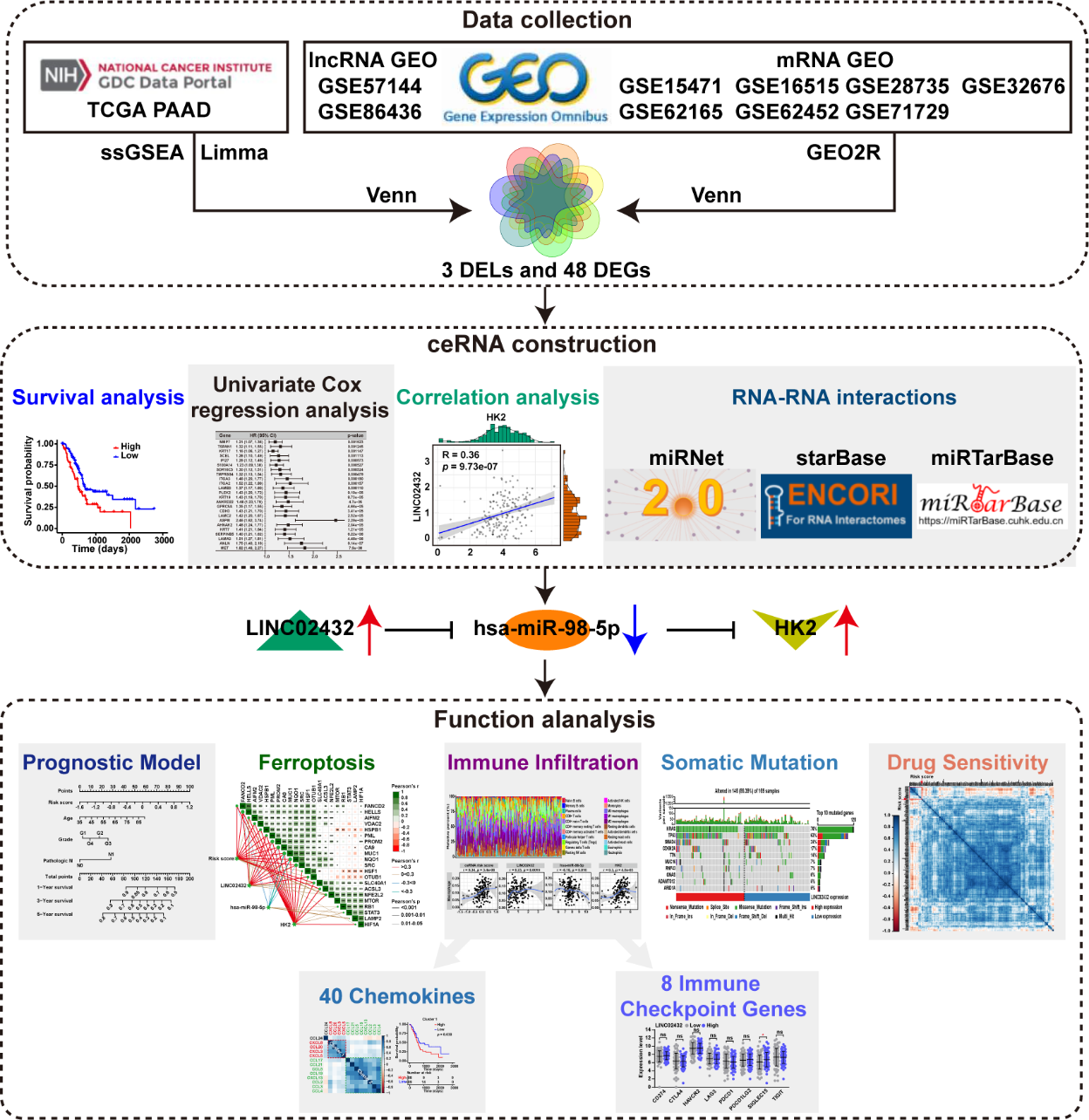
**

**Supplementary Figure 1 |** Flowchart of this research.

**
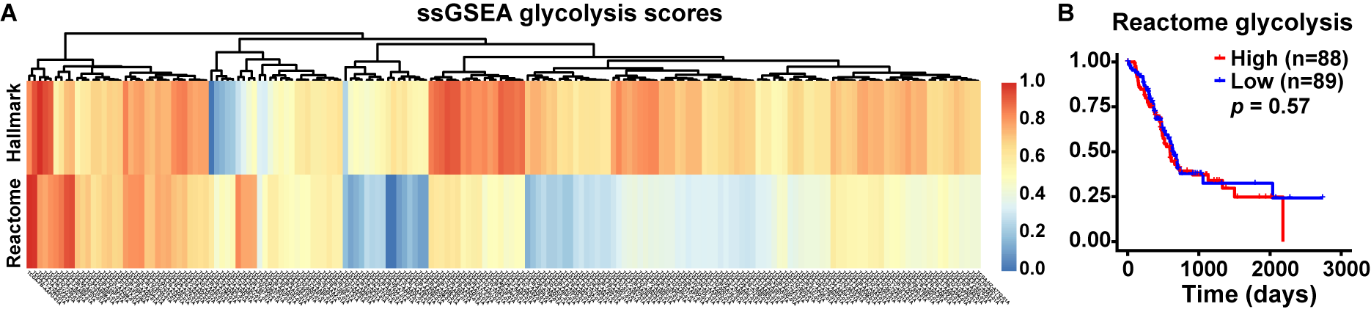
**

**Supplementary Figure 2 |** ssGSEA analysis of glycolysis pathway. **(A)** Heatmap of the ssGSEA score calculated by glycolysis pathway (hallmark glycolysis and reactome glycolysis). **(B)** Kaplan-Meier survival analysis of PAAD patients based on reactome glycolysis score.

**
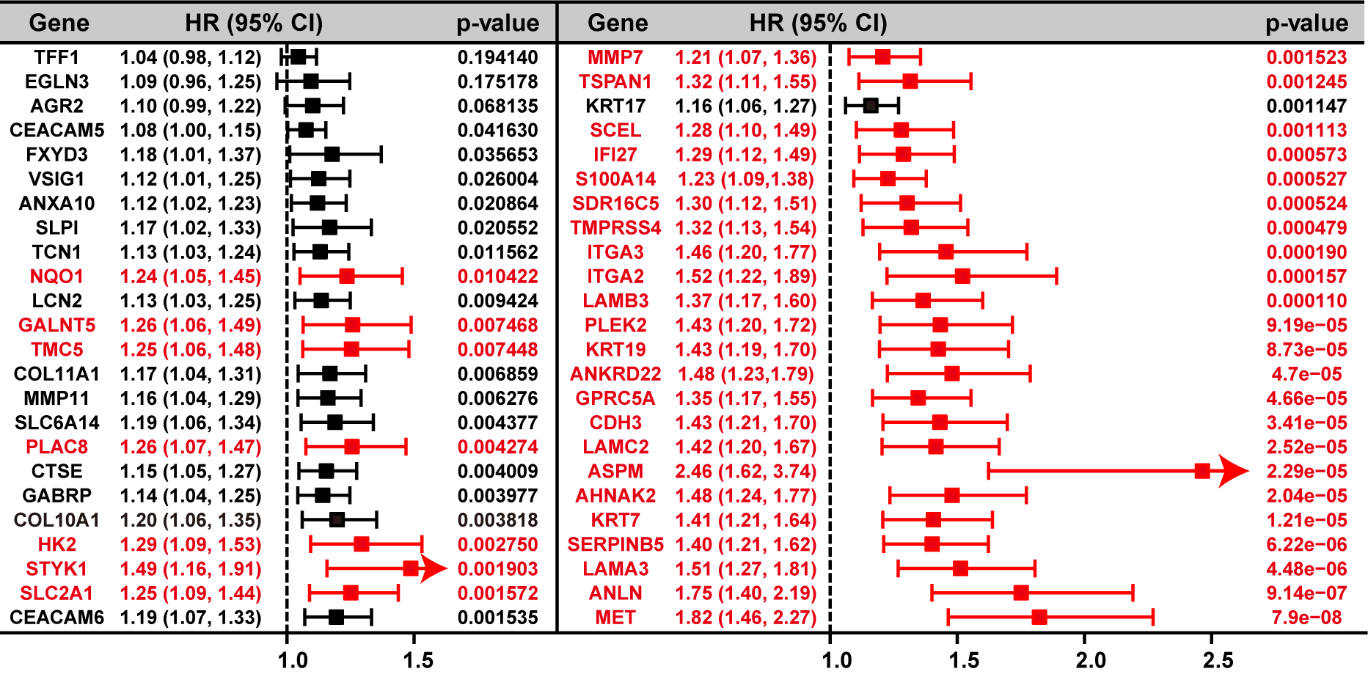
**

**Supplementary Figure 3 |** Univariate Cox regression analysis of 48 glycolysis-related genes. The red font indicated the key prognostic genes of glycolysis-related (p-value < 0.05 and HR > 1.2).


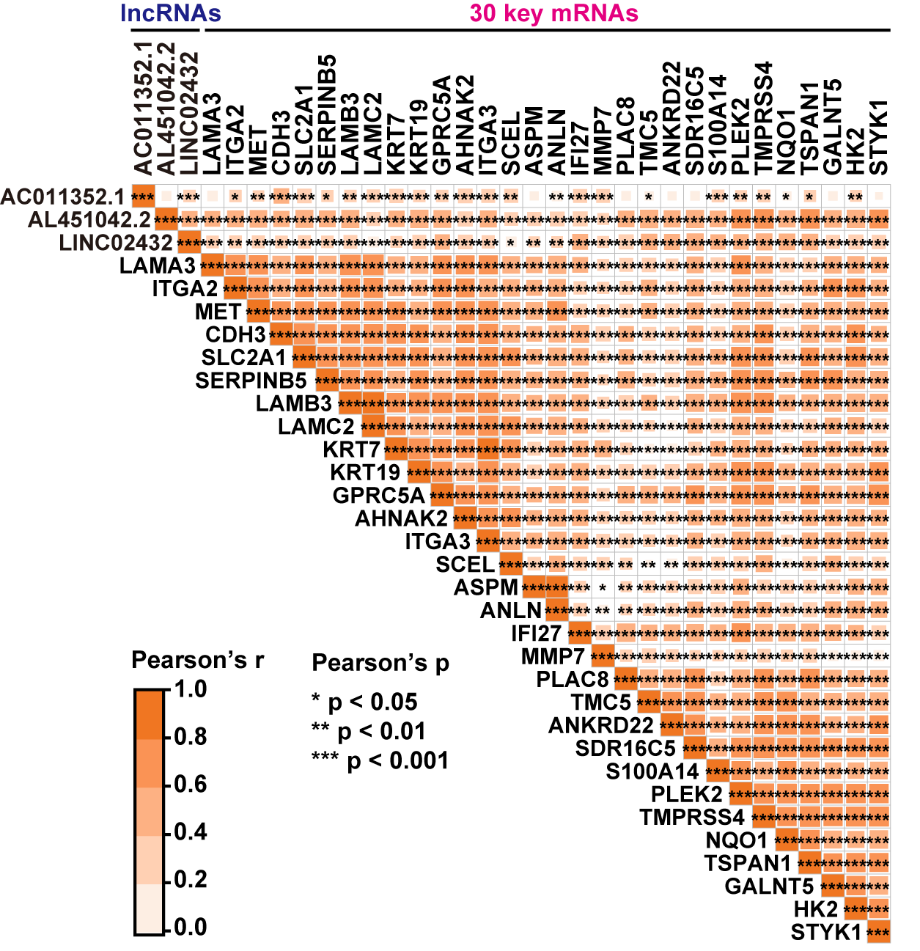


**Supplementary Figure 4 |** The Pearson correlation analysis was performed to determine the Pearson product moment correlation coefficient and significant differences between 3 lncRNAs and 30 key mRNAs.


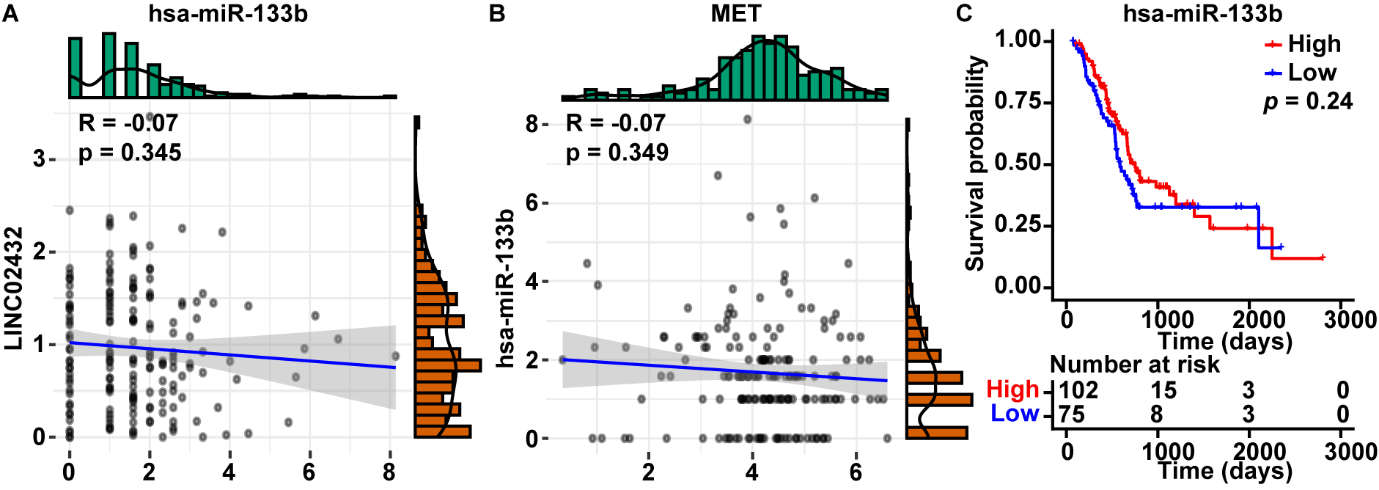


**Supplementary Figure 5 |** Correlation analysis and prognostic analysis. **(A)** The Pearson correlation analysis was used to determine the correlations between LINC02432 and hsa-miR-133b. **(B)** The correlation between hsa-miR-133b and MET was analyzed by Pearson correlation analysis. **(C)** Survival analysis of hsa-miR-133b was performed using Kaplan-Meier survival curves.


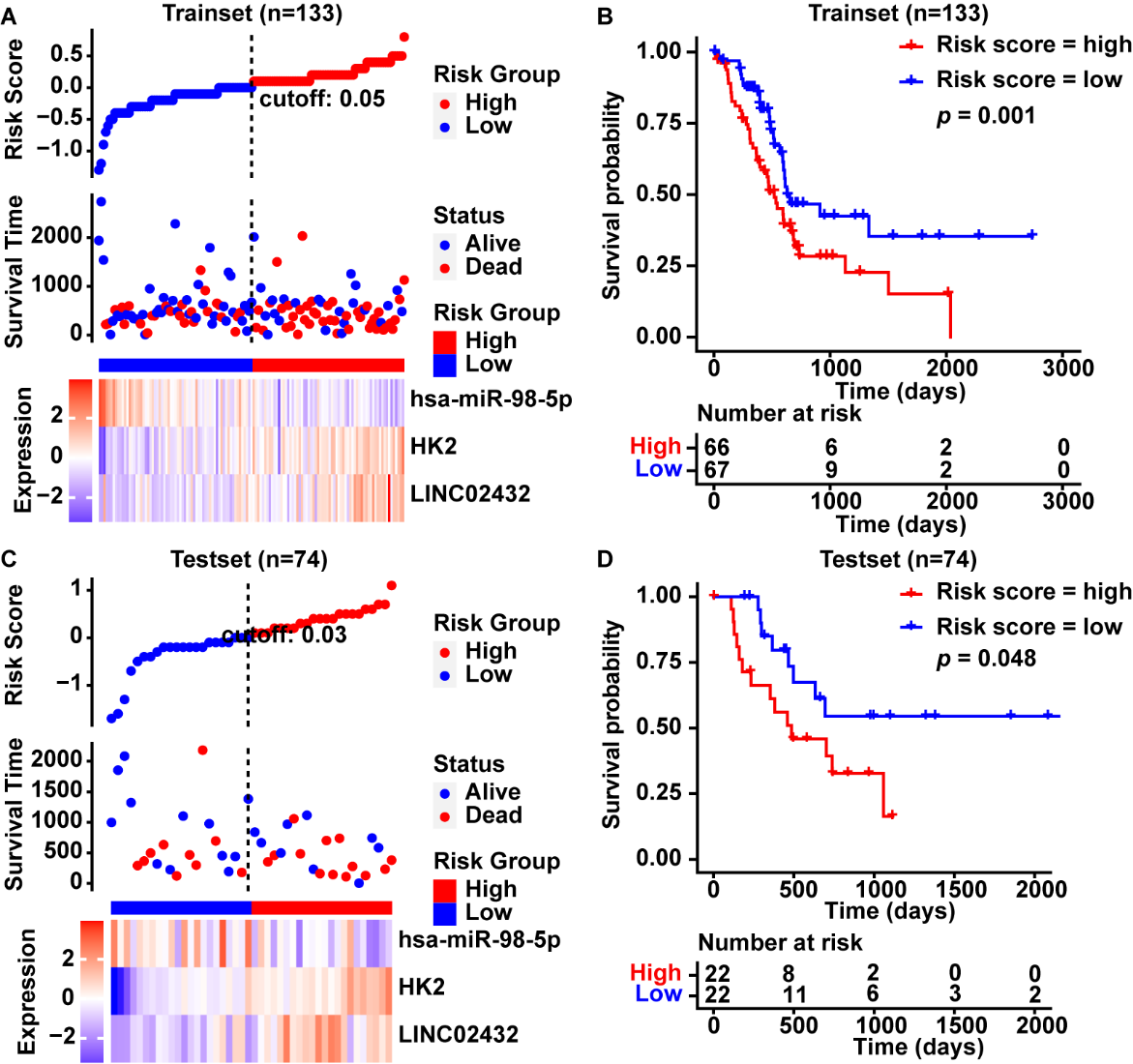


**Supplementary Figure 6 |** Comprehensive prognostic analysis of the three-gene signature in the TCGA PAAD. **(A)** The risk curves and scatterplots were performed in training cohort. **(B)** Kaplan-Meier curve analysis were performed in training cohort. **(C)** The risk curves and scatterplots were performed in testing cohort. **(D)** Kaplan-Meier curve analysis were performed in testing cohort.

**
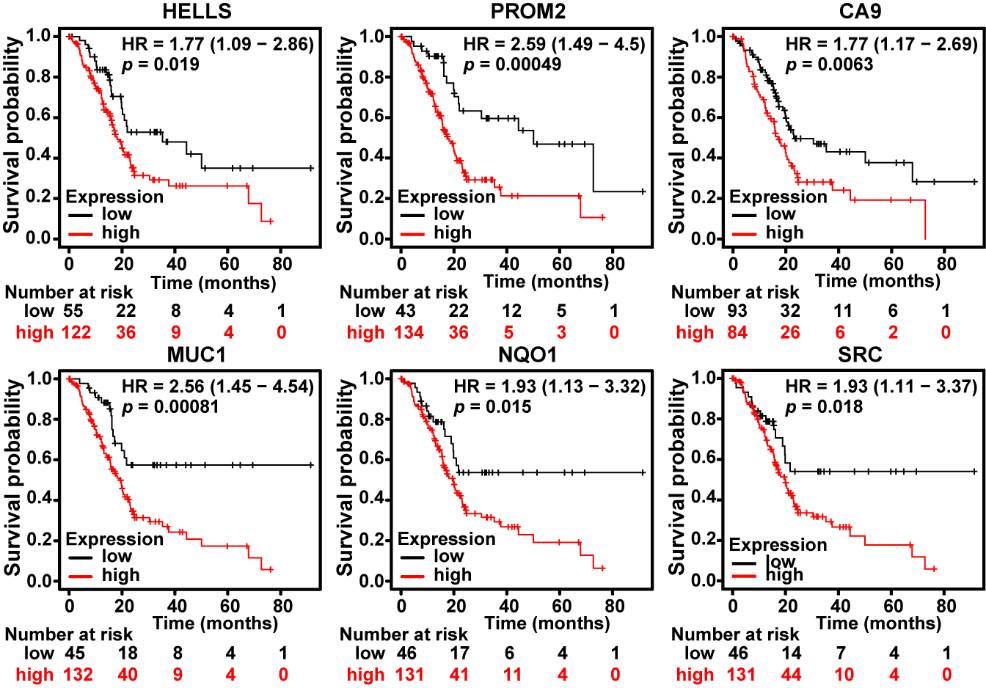
**

**Supplementary Figure 7 |** Kaplan-Meier survival analysis of HELLS, PROM2, CA9, MUC1, NQO1, and SRC expression from the Kaplan-Meier plotter database


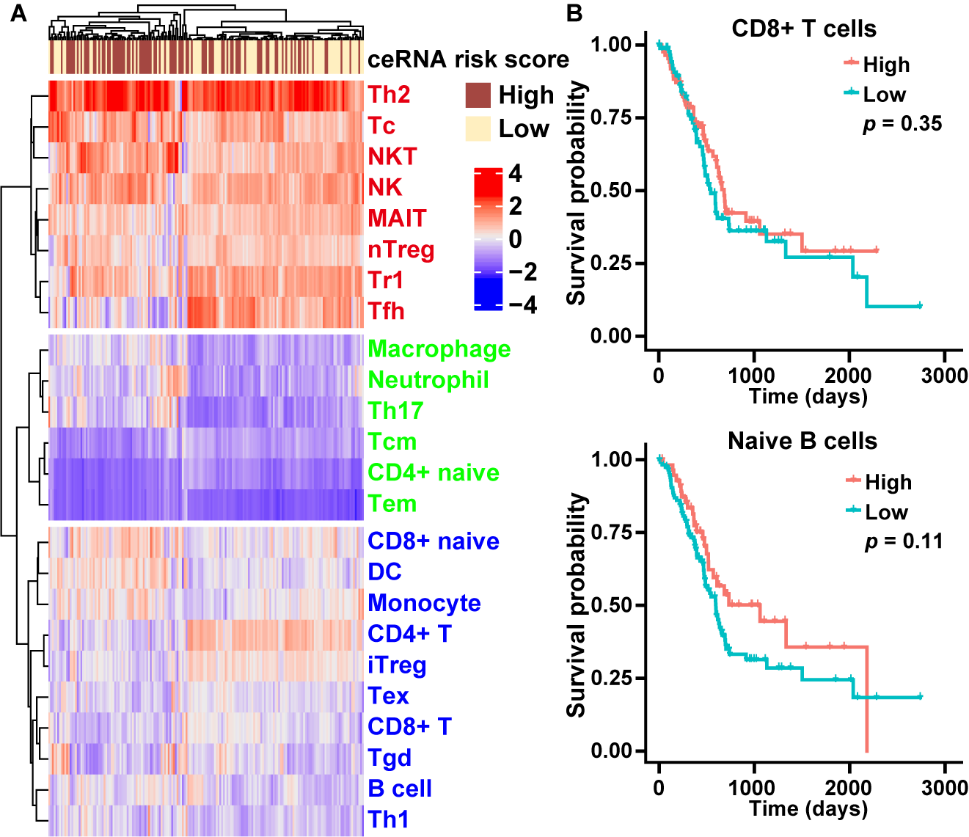


**Supplementary Figure 8 |** Tumor-infiltrating immune analysis. **(A)** ImmuCellAI estimated the relative abundance of 24 tumor-infiltrating immune cells. **(B)** Kaplan-Meier curves of CD8+ T cells, and Naive B cells for overall survival in PAAD patients.
